# Supplementary material for: MEK inhibitors activate Wnt signalling and induce stem cell plasticity in colorectal cancer
Source: Nat Commun. 2019 May 16;10:2197. doi: 10.1038/s41467-019-09898-0 (PMC6522484; doi:10.1038/s41467-019-09898-0)
Supplement: Supplementary file 1 — Supplementary Information [file 41467_2019_9898_MOESM1_ESM.pdf]

**Supplementary Information**

# MEK inhibitors activate Wnt signalling and induce stem cell plasticity in colorectal cancer

Zhan et al.

# Supplementary Figures

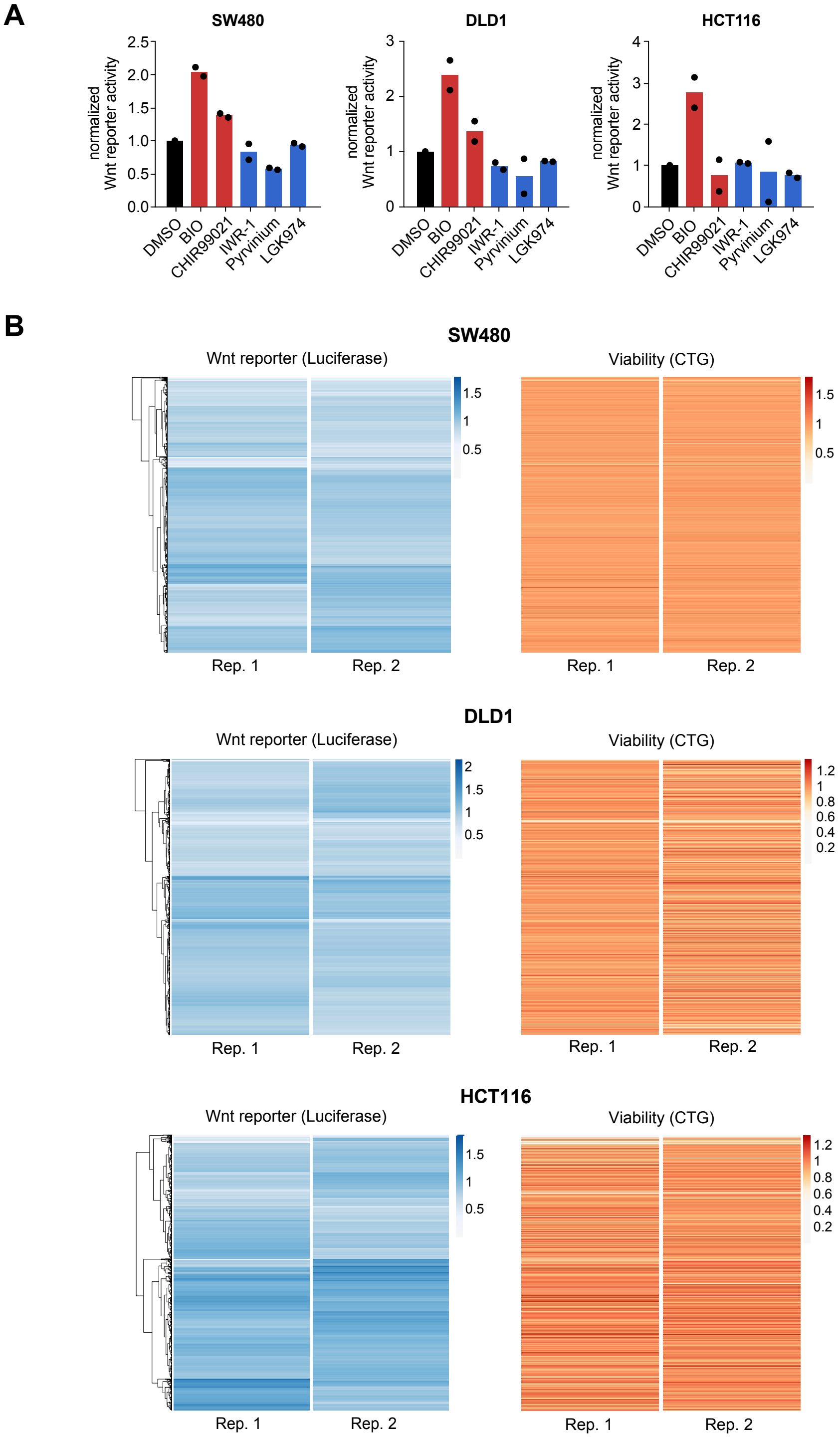

**Supplementary Figure 1: Pretests and extended results of the exploratory compound screen for modifiers of Wnt signaling.**

(A) Performance of small molecule Wnt activators and inhibitors in reporter cell lines. Wnt reporter cell lines SW480-7TFP, DLD1-7TFP and HCT116-TCF4 were treated with the GSK3B inhibitors BIO and CHIR99021, the tankyrase inhibitor IWR-1, the casein kinase 1 alpha activator pyrvinium and the porcupine inhibitor LGK974 at a concentration of 10 µM for 24 h, followed by measurement of cell viability and Wnt reporter activity. Data from two independent experiments are presented.

(B) Heatmaps showing TCF/Wnt and viability reporter signals of large exploratory compound screens. The heat maps represent the luciferase (Wnt, left map) and CTG (viability, right map) signals after normalization to the median signal of all samples of the respective plates. Results of both independent biological replicates are shown.

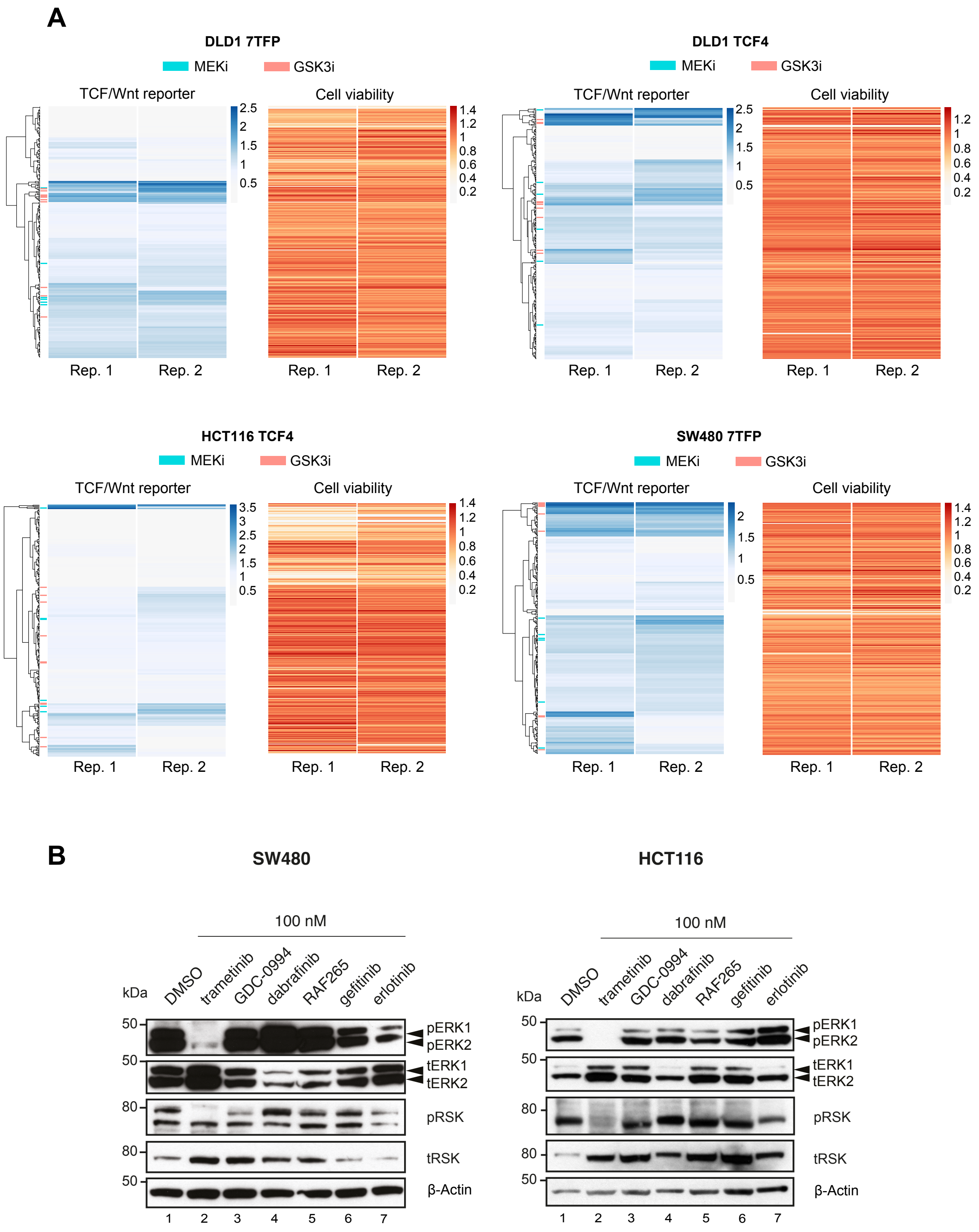
A
DLD1 7TFP
MEKi
GSK3i
TCF/Wnt reporter
Cell viability
Rep. 1
Rep. 2
DLD1 TCF4
HCT116 TCF4
SW480 7TFP
B
SW480
HCT116
100 nM
kDa
DMSO
trametinib
GDC-0994
dabrafinib
RAF265
gefitinib
erlotinib
pERK1
pERK2
tERK1
tERK2
pRSK
tRSK
β-Actin
1 2 3 4 5 6 7

**Supplementary Figure 2: Extended results of the kinase inhibitor screen for modifiers of Wnt signaling**

A) Heatmaps showing TCF/Wnt and viability reporter signals of focused kinase inhibitor screens. The heat maps represent the luciferase (Wnt, left map) and CTG (viability, right map) signals after normalization to the median signal of all samples of the respective plates. Results of both independent biological replicates are shown.

(B) Efficiency of different RAS pathway inhibitors on reducing ERK and RSK phosphorylation. SW480 and HCT116 cells were treated for 24 h with 100 nM of the MEK inhibitor trametinib, the ERK inhibitor GDC-0994, the RAF1/BRAF inhibitors dabrafenib and RAF265 and the EGFR inhibitors gefitinib and erlotinib for 24 h. Cells were then lysed for Western Blot analysis. A representative image of three independent biological replicates is shown.

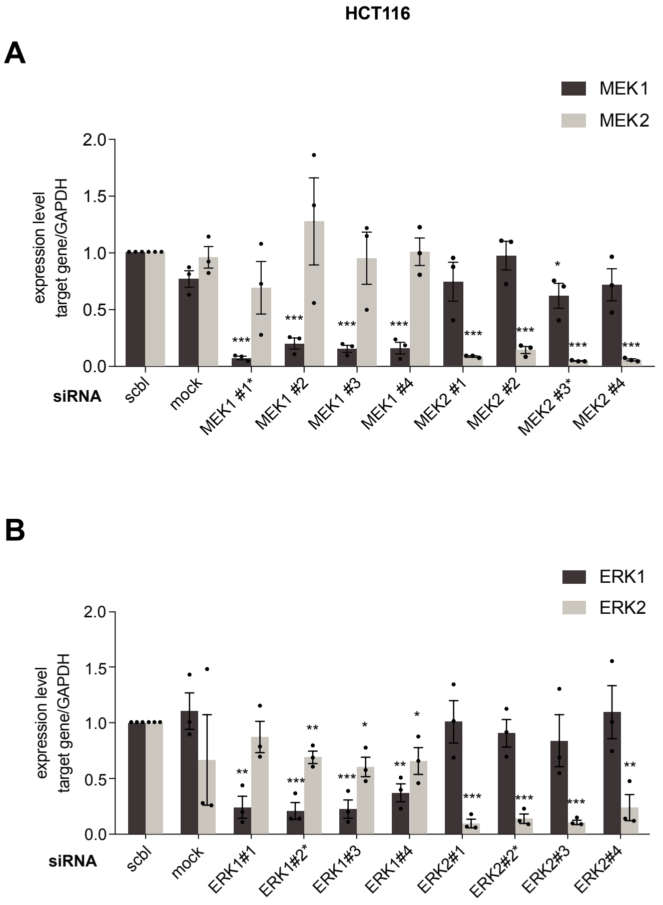


**Supplementary Figure 3: Performance of individual siRNAs targeting MEK1/2 and ERK1/2.**

(A-B) Efficiency of individual siRNAs targeting MEK1/2 (A) and ERK1/2 (B). HCT116 cells were transfected with the indicated siRNAs for 72 h, followed by measurement of expression levels of target gene by qPCR. siRNA selected for combinatorial knockdown are marked by (*).

(A-B) Data from three independent experiments are presented as mean ± s.e.m. ** $p < 0.01$, *** $p < 0.001$, two-sided Student's t-test.

A

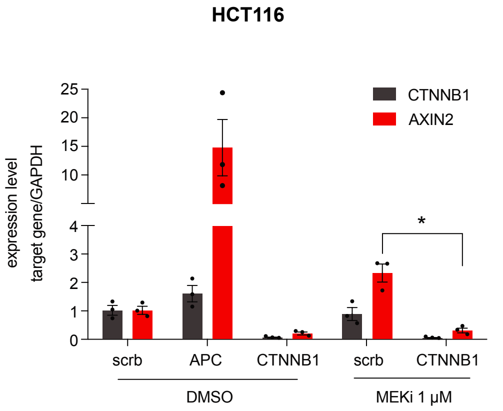


B

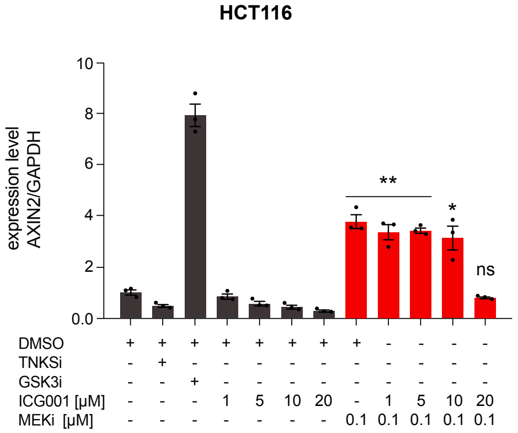


**Supplementary Figure 4: MEK inhibitor induced Wnt activation is dependent on beta-catenin.**

(A) siRNA mediated knockdown of beta-catenin (CTNNB1) abolishes the effect of MEK inhibition on Wnt activity. HCT116 cells were transfected with the indicated siRNAs for 48 h, followed by 24 h of incubation with trametinib. Expression levels of target genes were measured by qPCR.

(B) Pharmacological inhibition of CTNNB1/CBP interaction abolishes the effect of MEK inhibition on Wnt activity. HCT116 cells were treated for 24 h with the MEK inhibitor trametinib alone or together with different concentrations of ICG-001, a small molecule inhibitor of CTNNB1/CBP interaction. *AXIN2* expression was measured by qPCR. At high concentrations of ICG-001, the stimulating effect of trametinib on *AXIN2* expression is abolished.

(A-B) Data from three independent experiments are presented as mean ± s.e.m. * $p < 0.05$, ** $p < 0.01$, two-sided Student's t-test.

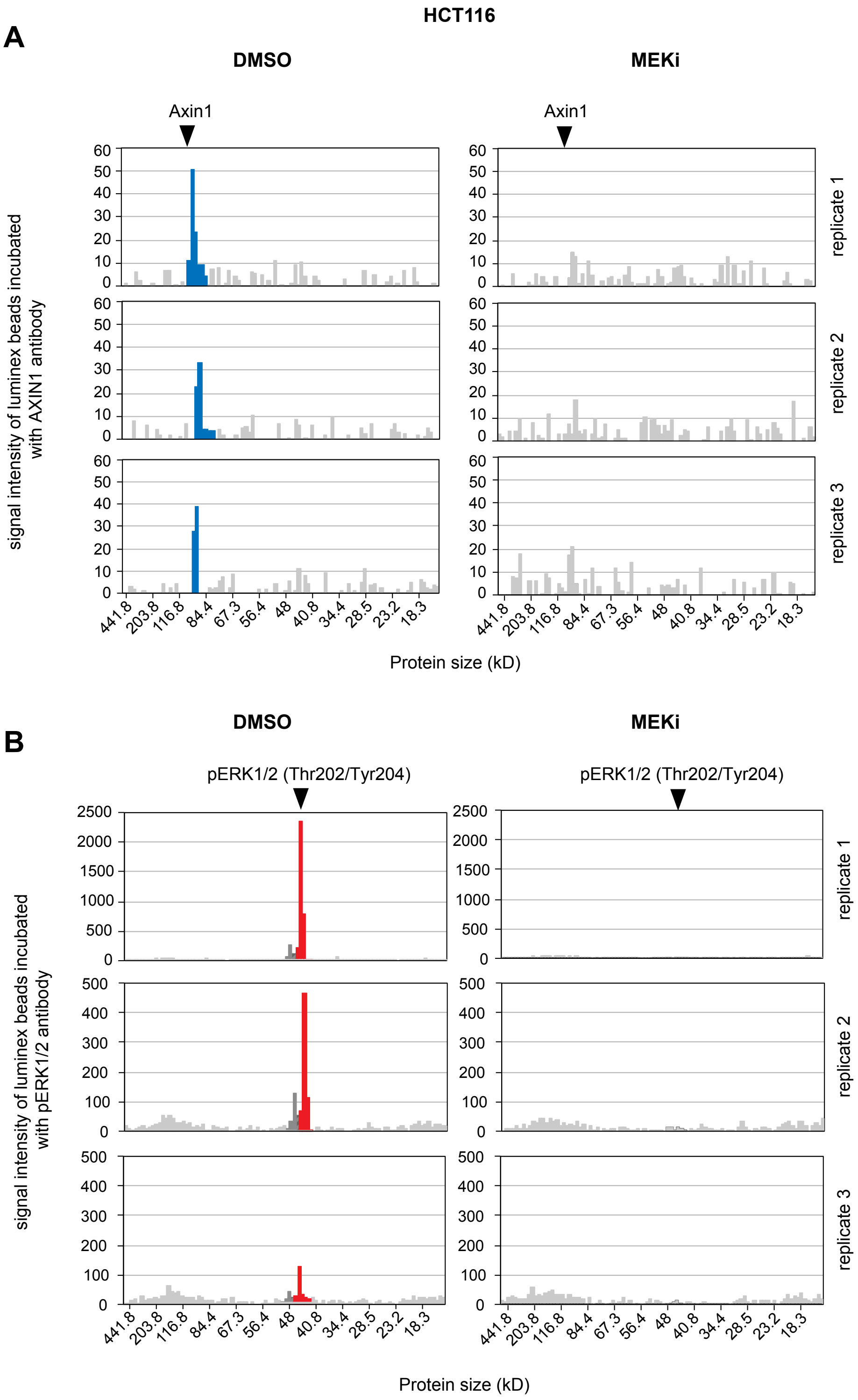

HCT116
A
DMSO
MEKi
Axin1
Axin1
60
50
40
30
20
10
0
replicate 1
replicate 2
replicate 3
signal intensity of luminex beads incubated with AXIN1 antibody
441.8
203.8
116.8
84.4
67.3
56.4
48
40.8
34.4
28.5
23.2
18.3
Protein size (kD)
B
DMSO
MEKi
pERK1/2 (Thr202/Tyr204)
pERK1/2 (Thr202/Tyr204)
2500
2000
1500
1000
500
400
300
200
100
0
signal intensity of luminex beads incubated with pERK1/2 antibody
Protein size (kD)

**Supplementary Figure 5: DigiWest demonstrates reduction of AXIN1 protein levels upon trametinib treatment.**

(A) HCT116 cells were treated for 24 h with 1 μM of trametinib and then lysed and processed for DigiWest analysis. The signal intensity of Luminex beads incubated with an anti-AXIN1 (blue) and anti-phosphoERK1/2 (Thr202/Tyr204) antibody (red) is shown for all three biological replicates.

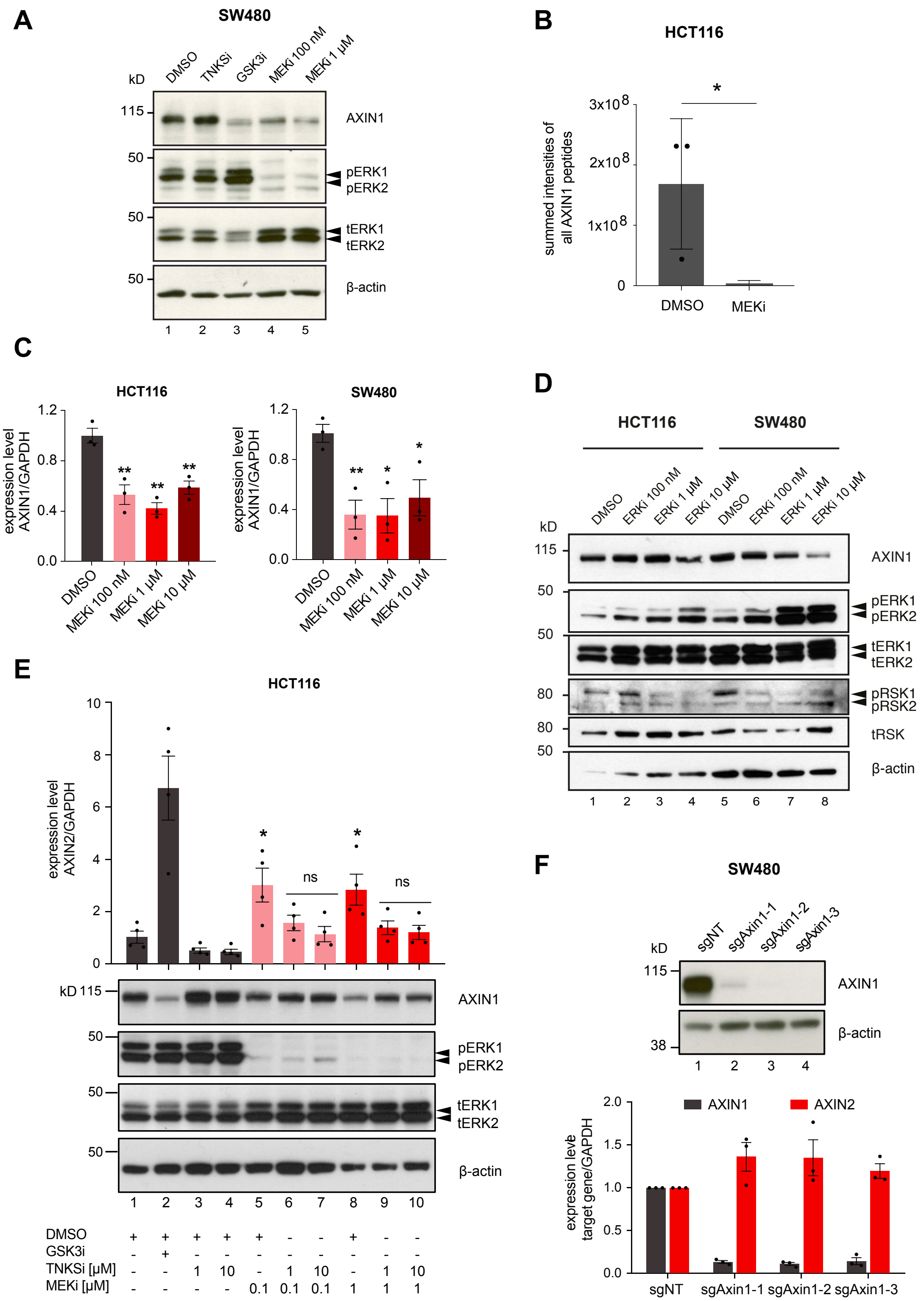

A
SW480
kD
DMSO
TNKSi
GSK3i
MEKi 100 nM
MEKi 1 μM
115
50
50
50
AXIN1
pERK1
pERK2
tERK1
tERK2
β-actin
1 2 3 4 5
B
HCT116
summed intensities of all AXIN1 peptides
3x10^8
2x10^8
1x10^8
0
*
DMSO
MEKi
C
HCT116
SW480
expression level AXIN1/GAPDH
1.2
0.8
0.4
0.0
**
*
DMSO
MEKi 100 nM
MEKi 1 μM
MEKi 10 μM
D
HCT116
SW480
kD
DMSO
ERKi 100 nM
ERKi 1 μM
ERKi 10 μM
115
50
80
AXIN1
pERK1
pERK2
tERK1
tERK2
pRSK1
pRSK2
tRSK
β-actin
1 2 3 4 5 6 7 8
E
HCT116
expression level AXIN2/GAPDH
10
8
6
4
2
0
*
ns
kD 115
50
AXIN1
pERK1
pERK2
tERK1
tERK2
β-actin
1 2 3 4 5 6 7 8 9 10
DMSO + + + + + - - + - -
GSK3i - + - - - - - - - -
TNKSi [μM] - - 1 10 - 1 10 - 1 10
MEKi [μM] - - - - 0.1 0.1 0.1 1 1 1
F
SW480
kD
sgNT
sgAxin1-1
sgAxin1-2
sgAxin1-3
115
38
AXIN1
β-actin
1 2 3 4
AXIN1
AXIN2
expression level target gene/GAPDH
2.0
1.5
1.0
0.5
0.0
sgNT
sgAxin1-1
sgAxin1-2
sgAxin1-3

**Supplementary Figure 6: AXIN1 protein levels determine the effect of trametinib on Wnt signaling.**

(A) Western Blot showing reduction of AXIN1 protein levels in SW480 cells upon MEK inhibition. Colorectal cancer cells were treated for 24 h with the indicated compounds and then lysed for Western Blot analysis. A representative image of three independent biological replicates is shown.

(B) Affinity purification of AXIN1 and subsequent mass spectrometry analysis reveals an approximately 50-fold reduction in AXIN1 abundance upon treatment with trametinib. Data from three independent experiments are presented as mean ± s.e.m. * $p < 0.05$, one-sided Student's t-test.

(C) Trametinib causes a reduction of *AXIN1* expression. SW480 and HCT116 cells were treated for 24 h with increasing concentrations of trametinib and expression of *AXIN1* and *GAPDH* were determined by qPCR.

(D) Western Blot showing downregulation of AXIN1 protein levels in HCT116 and SW480 cells upon ERK inhibition. Colorectal cancer cell lines were treated for 24 h with the indicated compounds and then lysed for Western Blot analysis. A representative image of three independent biological replicates is shown.

(E) Stabilization of AXIN1 by treatment with tankyrase inhibitor XAV939 abolishes MEK inhibitor induced Wnt activation. HCT116 cells were co-treated with different concentrations of trametinib and XAV939. Western Blot analysis shows that protein levels of *AXIN1* was reduced by trametinib treatment, but restored by co-treatment with XAV939. Treatment with trametinib alone increased *AXIN2* expression, but co-treatment with XAV939 abolished this effect. A representative image of three independent Western Blot replicates is shown.

(F) CRISPRi mediated knockdown of *AXIN1* mildly stimulates *AXIN2* expression in SW480. SW480 cells stably expressing dCas9-KRAB and sgRNAs targeting *AXIN1* were generated by lentiviral infection and antibiotic selection. Expression levels of *AXIN1* and *AXIN2* were determined by qPCR. Efficiency of knockdown was additionally measured by Western Blot.

(B-D) Data from three (C, F) or four (E) independent experiments are presented as mean ± s.e.m. * $p < 0.05$, ** $p < 0.01$, *** $p < 0.001$, two-sided Student's t-test.

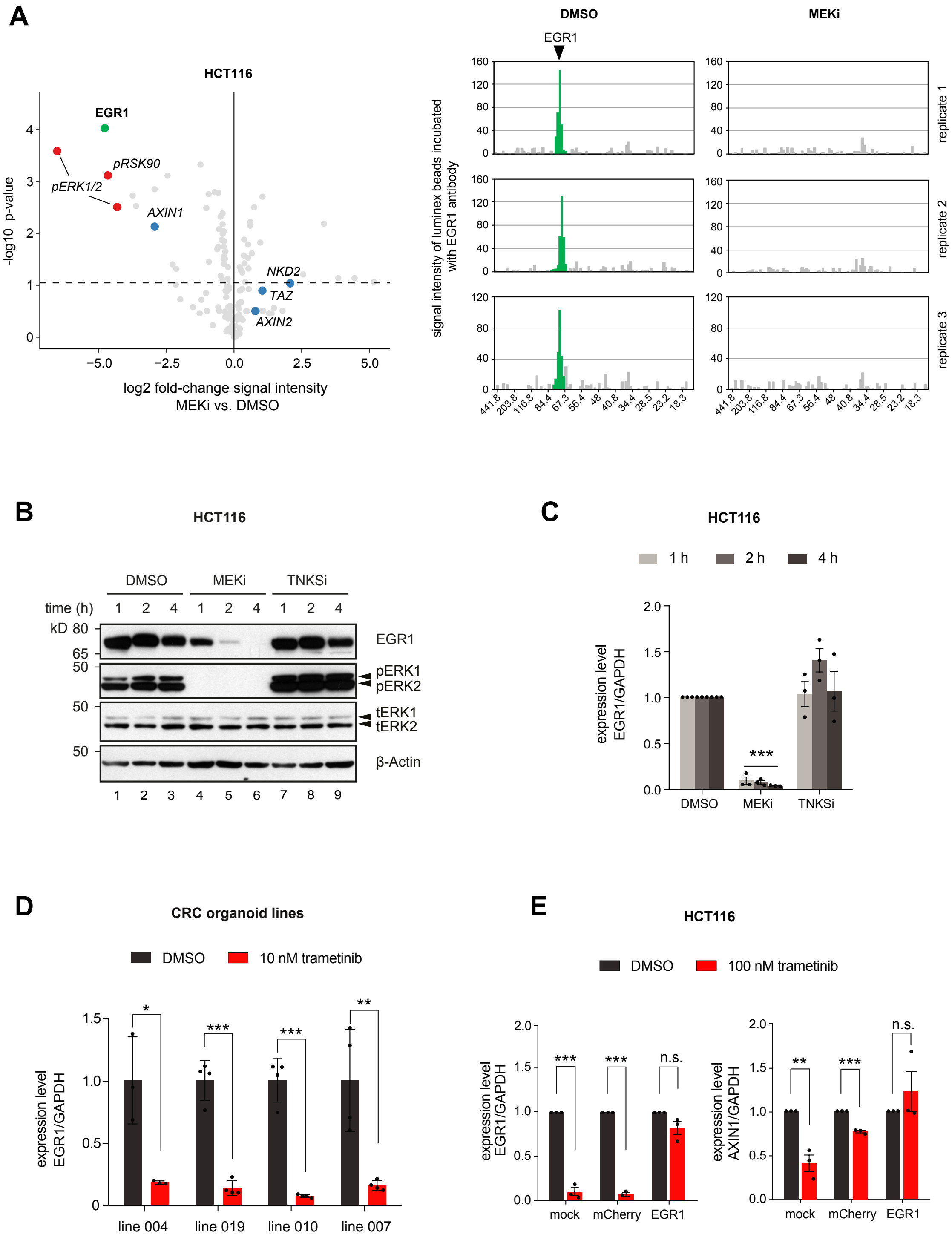
A
HCT116
EGR1
pRSK90
pERK1/2
AXIN1
NKD2
TAZ
AXIN2
-log10 p-value
log2 fold-change signal intensity
MEKi vs. DMSO
DMSO
MEKi
signal intensity of luminex beads incubated with EGR1 antibody
replicate 1
replicate 2
replicate 3
B
HCT116
DMSO
MEKi
TNKSi
time (h)
kD
pERK1
pERK2
tERK1
tERK2
β-Actin
C
HCT116
1 h
2 h
4 h
expression level EGR1/GAPDH
***
D
CRC organoid lines
DMSO
10 nM trametinib
line 004
line 019
line 010
line 007
E
HCT116
100 nM trametinib
mock
mCherry
n.s.
expression level AXIN1/GAPDH

**Supplementary Figure 7: Trametinib causes rapid downregulation of EGR1 levels in CRC.**

(A) Volcano plot showing signal intensity of EGR1 (green) and selected Wnt and Ras pathway members in trametinib versus DMSO treated HCT116 cells as determined by DigiWest analysis. Mean values of three independent replicates are shown. Statistical significance was determined by a moderated t-test. (B) The signal intensity of Luminex beads incubated with an anti-EGR1 antibody (green) is shown for all three biological replicates.

(C-D) MEK inhibition induces a rapid depletion of EGR1 protein levels and expression. Trametinib (100 nM), XAX939 (10 µM) and DMSO were added to cells, followed by parallel determination of protein (C) and transcript levels (D) at different time points (1, 2 and 4 h). Representative image of three biological replicates is presented in (C). Data from four independent experiments are presented as mean ± s.e.m. * $p < 0.05$, ** $p < 0.01$, *** $p < 0.001$, two-sided Student's t-test.

(E) MEK inhibition downregulates expression of *EGR1* in colorectal cancer organoids. Four colorectal cancer organoid lines were treated for 72 h with the 10 nM trametinib or DMSO and the relative expression of *EGR1* was measured using qPCR. Data from at three (organoid line 004) or four (organoid line 007, 015, 019) independent experiments are presented as mean ± s.e.m. * $p < 0.05$, ** $p < 0.01$, *** $p < 0.001$, two-sided Student's t-test.

(F) Overexpression of *EGR1* rescues downregulation of *AXIN1* transcript levels by MEK inhibition. HCT116 cells were either mock transfected or transfected with plasmids encoding mCherry or EGR1. Twenty-four hours after transfection, cells were treated with either DMSO or 100 nM trametinib for 4 h. Cells were then harvested and expression levels of *AXIN1* and *EGR1* determined by qPCR. Data from three independent experiments are presented as mean ± s.e.m. ** $p < 0.01$ and *** $p < 0.001$, two-sided Student's t-test.

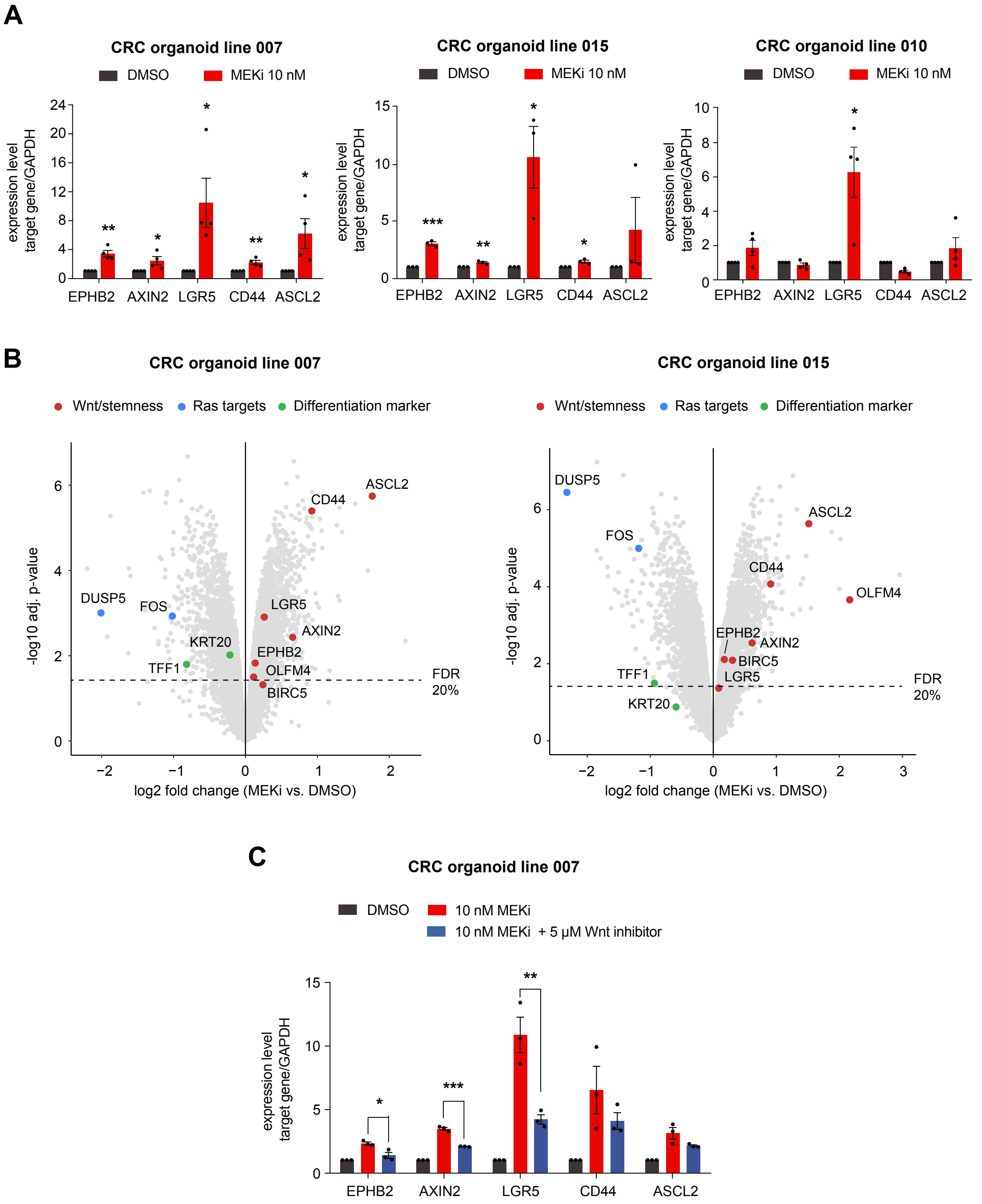
A
CRC organoid line 007
DMSO
MEKi 10 nM
expression level
target gene/GAPDH
0
4
8
12
16
20
24
**
*
*
**
*
EPHB2
AXIN2
LGR5
CD44
ASCL2
CRC organoid line 015
DMSO
MEKi 10 nM
expression level
target gene/GAPDH
0
5
10
15
***
**
*
*
EPHB2
AXIN2
LGR5
CD44
ASCL2
CRC organoid line 010
DMSO
MEKi 10 nM
expression level
target gene/GAPDH
0
2
4
6
8
10
*
EPHB2
AXIN2
LGR5
CD44
ASCL2
B
CRC organoid line 007
Wnt/stemness
Ras targets
Differentiation marker
-log10 adj. p-value
0
2
4
6
ASCL2
CD44
DUSP5
FOS
LGR5
AXIN2
KRT20
EPHB2
TFF1
OLFM4
BIRC5
FDR
20%
−2
−1
0
1
2
log2 fold change (MEKi vs. DMSO)
CRC organoid line 015
Wnt/stemness
Ras targets
Differentiation marker
-log10 adj. p-value
0
2
4
6
DUSP5
FOS
ASCL2
CD44
OLFM4
EPHB2
AXIN2
BIRC5
TFF1
LGR5
KRT20
FDR
20%
−2
−1
0
1
2
3
log2 fold change (MEKi vs. DMSO)
C
CRC organoid line 007
DMSO
10 nM MEKi
10 nM MEKi + 5 µM Wnt inhibitor
expression level
target gene/GAPDH
0
5
10
15
*
***
**
EPHB2
AXIN2
LGR5
CD44
ASCL2

**Supplementary Figure 8: Trametinib induces Wnt activity in CRC organoids.**

(A-B) MEK inhibition induces expression of Wnt target genes and intestinal stemness markers. (A) Three CRC organoid lines were treated for 72 h with the 10 nM trametinib or DMSO and the expression of selected genes was measured using qPCR. Data from three (organoid line 007, 010) or four (organoid line 015) independent experiments are presented as mean ± s.e.m. * $p < 0.05$, ** $p < 0.01$, *** $p < 0.001$, two-sided Student's t-test. (B) Expression analysis with microarray of two colon cancer organoid lines treated with trametinib shows increased expression of Wnt target genes and intestinal stemness markers, and a reduction of RAS target genes and differentiation markers.

(C) Human colorectal cancer organoids were treated with 10 nM of trametinib and 5μM of the Wnt inhibitor PRI-724. Expression of selected Wnt target and stemness genes was analysed by qPCR. Data from three independent experiments are presented as mean ± s.e.m. * $p < 0.05$, ** $p < 0.01$, *** $p < 0.001$, two-sided Student's t-test.

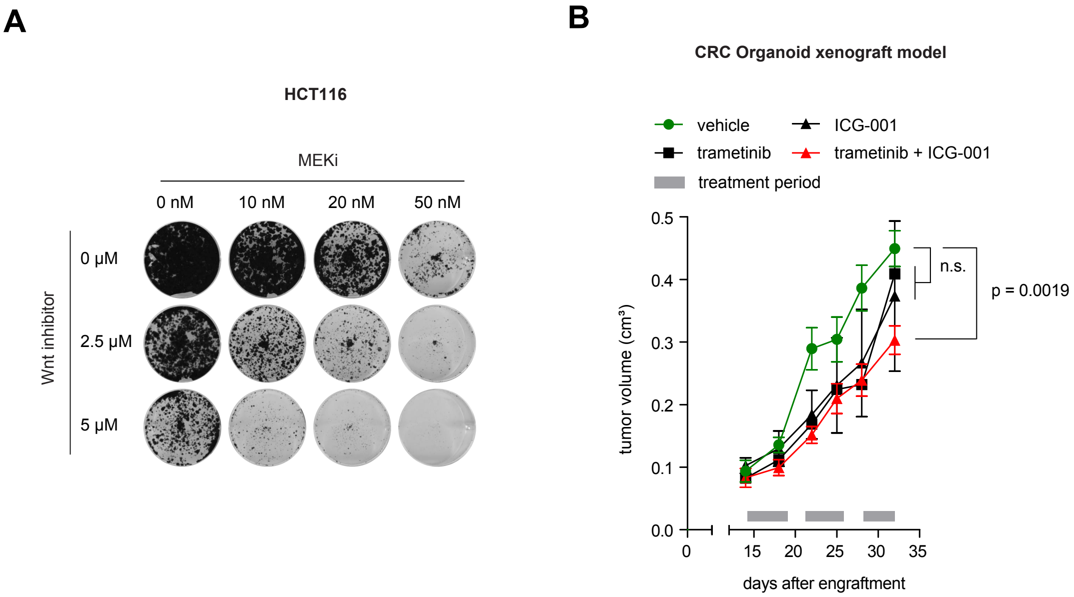


**Supplementary Figure 9: Co-inhibition of Wnt and Ras signalling reduces proliferation of CRC.**

(A) Co-treatment of trametinib with PRI-724 synergistically reduces proliferation of colorectal cancer cells. HCT116 were seeded at low density and then treated for six days with combinations of trametinib and PRI-724. Cells were then allowed to recover for four days in media without drugs before fixation and staining with crystal violet solution. A representative image of three independent experiments is shown.

(B) Combined MEK and Wnt inhibition is effective in reducing cancer cell proliferation in a PDX model. Colorectal cancer organoids were engrafted into NOD/SCID gamma mice by subcutaneous injections. Two weeks after engraftment, tumors were treated with trametinib (1 mg kg^$^{-1}$ body weight), ICG-001 (200 mg kg^$^{-1}$ body weight), a combination of both substances or vehicle solution once daily, for a total of 14 d. Seven mice were treated in each group and indicated levels of significance were determined by a two-sided Student's t-test.

## Supplementary Tables

**Supplementary Table 1: Antibodies used for the DigiWest assays.** For antibodies that produce two characteristic signal peaks, both peaks are separately enlisted.

| Antibody | Company | Catalogue # | Species |
|---|---|---|---|
| Actin | Santa Cruz | sc-1616 | goat |
| A-Raf | Cell Signaling Technology | 4432 | rabbit |
| A-Raf - phospho - Ser299 | Cell Signaling Technology | 4431 | rabbit |
| A-Raf - phospho - Tyr301/Tyr302 | Biorbyt | orb5910 | rabbit |
| ASCL2/Mash2 | R&D | AF6539 | sheep |
| Axin1 | Cell Signaling Technology | 3323 | rabbit |
| Axin2 (conduction) | Cell Signaling Technology | 2151 | rabbit |
| Bcl9 | ABNOVA | H00000607-M01 | mouse |
| beta-catenin | Cell Signaling Technology | 8480 | rabbit |
| beta-Catenin - phospho - Ser552 | Cell Signaling Technology | 9566 | rabbit |
| beta-Catenin - phospho - Ser675 | Cell Signaling Technology | 9567 | rabbit |
| beta-Catenin (non-pospho Ser33/37/Thr41; active) | Cell Signaling Technology | 8814 | rabbit |
| Bmi1 | Cell Signaling Technology | 6964 | rabbit |
| BMP4 | Epitomics | 5163-1 | rabbit |
| b-Raf - phospho - Ser445 | Cell Signaling Technology | 2696 | rabbit |
| C/EBP alpha - Peak 1 | Cell Signaling Technology | 8178 | rabbit |
| C/EBP alpha - Peak 2 | Cell Signaling Technology | 8178 | rabbit |
| C/EBP beta | Cell Signaling Technology | 3087 | rabbit |
| C/EBP beta - phospho - Thr235 | Cell Signaling Technology | 3084 | rabbit |
| Casein kinase 1 alpha | Cell Signaling Technology | 2655 | rabbit |
| Casein kinase 1 delta | Abcam | ab85320 | mouse |
| Caseinkinase 1 epsilon | Nanotools | 0172-100 | mouse |
| Caseinkinase 2 alpha | Cell Signaling Technology | 2656 | rabbit |
| CBP | Cell Signaling Technology | 7389 | rabbit |

| | | | |
|---|---|---|---|
| CD133 | Cell Signaling Technology | 3663 | rabbit |
| Cerberus | Santa Cruz | sc-15132 | goat |
| cJun | Cell Signaling Technology | 9165S | rabbit |
| cJun – phosphor- Ser63 | Cell Signaling Technology | 2361S | rabbit |
| c-Raf | Cell Signaling Technology | 9422 | rabbit |
| c-Raf - phospho - Ser259 | Cell Signaling Technology | 9421 | rabbit |
| c-Raf - phospho - Ser338 | Cell Signaling Technology | 9427 | rabbit |
| DKK2 | Cell Signaling Technology | 4683 | rabbit |
| DUSP10 (MKP-5) | Cell Signaling Technology | 3483 | rabbit |
| DUSP14 (MKP6, MKP-6, MKP-L) | ProteinTech Group | 10172-2-AP | rabbit |
| DUSP16 (MKP-7) | Abcam | ab65151 | rabbit |
| DUSP4 | Abcam | ab72593 | rabbit |
| DUSP6 (MKP3, MKP-3, PYST1) | ProteinTech Group | 10433-1-AP | rabbit |
| Dvl2 | Cell Signaling Technology | 3224 | rabbit |
| Dvl3 | Cell Signaling Technology | 3218 | rabbit |
| DVL3 (4D3) | Santa Cruz | sc-8027 | mouse |
| EGFR (ErB-1, HER1) - phospho - Tyr1173 | Cell Signaling Technology | 4407 | rabbit |
| EGFR (ErB-1, HER1) - phospho - Tyr845 | Cell Signaling Technology | 2231 | rabbit |
| EGR1 | Cell Signaling Technology | 4153 | rabbit |
| Elk-1 | Cell Signaling Technology | 9182 | rabbit |
| Erk1/2 (MAPK p44/42) - Peak 2 | Cell Signaling Technology | 4695 | rabbit |
| Erk1/2 (MAPK p44/42) - phospho - Thr202/Tyr204 - Peak 1 | Cell Signaling Technology | 4370B | rabbit |
| Erk1/2 (MAPK p44/42) - phospho - Thr202/Tyr204 - Peak 2 | Cell Signaling Technology | 4370B | rabbit |
| Erk2 (MAPK p42) | Cell Signaling Technology | 9108 | rabbit |
| Evi - Peak 1 | Protein Tech | 17950-1-AP | rabbit |
| Evi - Peak 2 | Protein Tech | 17950-1-AP | rabbit |
| FABP4 | Cell Signaling Technology | 3544 | rabbit |
| FRA1 | Cell Signaling Technology | 5281 | rabbit |

| | | | |
|---|---|---|---|
| FRA1 - phospho - Ser265 | Cell Signaling Technology | 5841 | rabbit |
| FRA2 | Abcam | ab124830 | rabbit |
| Frizzled 1 | Abcam | ab71342 | rabbit |
| Frizzled 7 | Abgent | AB64636 | rabbit |
| GAPDH | Abcam | ab9485 | rabbit |
| GSK3 alpha/beta - phospho - Ser21/Ser9 - Peak 1 | Cell Signaling Technology | 9331 | rabbit |
| GSK3 alpha/beta - phospho - Ser21/Ser9 - Peak 2 | Cell Signaling Technology | 9331 | rabbit |
| GSK3 alpha/beta - phospho - Tyr279/Tyr216 - Peak 1 | Abcam | ab68476 | rabbit |
| GSK3 alpha/beta - phospho - Tyr279/Tyr216 - Peak 2 | Abcam | ab68476 | rabbit |
| GSK3 beta | Cell Signaling Technology | 9315 | rabbit |
| GSK3 beta - phospho - Ser9 | Cell Signaling Technology | 9336 | rabbit |
| HRAS | Santa Cruz | sc-29 | mouse |
| Her2 | Dako | A0485 | rabbit |
| HIF1 beta (ARNT) | Cell Signaling Technology | 3718 | rabbit |
| Ki-67 | USBiological | K1700-05D | rabbit |
| KLF2 | Sigma | AV37859 | rabbit |
| KLF4 | Cell Signaling Technology | 12173S | rabbit |
| KSR1 - phospho - Ser392 | Cell Signaling Technology | 4951 | rabbit |
| LRP6 | Cell Signaling Technology | 3395 | rabbit |
| LRP6 – phospho – Ser1490 | Cell Signaling Technology | 2568S | rabbit |
| MEK1 | Cell Signaling Technology | 2352 | mouse |
| MEK1 - phospho - Thr286 | Epitomics | 2336-1 | rabbit |
| MEK1 - phospho - Thr292 | Epitomics | 2233-1 | rabbit |
| MEK1/2 - phospho - Ser217/Ser221 | Cell Signaling Technology | 9154 | rabbit |
| MEK2 | Cell Signaling Technology | 9125 | rabbit |
| MEKK3 | Transduction Laboratories | M79820 | mouse |
| MKK3/6– phospho- Ser189/Ser207 | Cell Signaling Technology | 9236 | rabbit |
| Mnk1 | Cell Signaling Technology | 2195 | rabbit |

| | | | |
|---|---|---|---|
| Mnk1 - phospho - Thr197/Thr202 | Cell Signaling Technology | 2111 | rabbit |
| MSK1 | Cell Signaling Technology | 3489 | rabbit |
| MSK1 - phospho – Thr589 | Cell Signaling Technology | 9595P | rabbit |
| Mst1 | Cell Signaling Technology | 3682 | rabbit |
| MVP (LRP) | Transduction Laboratories | L44820 | mouse |
| Naked 1 | Cell Signaling Technology | 2262 | rabbit |
| Nanog | Cell Signaling Technology | 4893 | rabbit |
| PCNA | Abcam | ab29 | mouse |
| PTPRR (PTP-SL, PTP13) | Sigma | AV45387 | rabbit |
| Ras | Cell Signaling Technology | 8955 | rabbit |
| RKIP (PBP, PEBP, PEBP1) | Cell Signaling Technology | 4742 | rabbit |
| RSK 1 (p90RSK) | Epitomics | 2004-1 | rabbit |
| RSK 1 (p90RSK) - phospho - Ser380 | Cell Signaling Technology | 9341 | rabbit |
| RSK 1 (p90RSK) - phospho - Thr573 | Abcam | ab62324 | rabbit |
| RSK 1/2/3 | Cell Signaling Technology | 9347 | rabbit |
| RSK 3 - phospho - Thr353/Thr356 | Epitomics | 2012-1 | rabbit |
| RSK 4 - phospho - Ser235 | Epitomics | 2151-1 | rabbit |
| SFRP2 | Abcam | ab111874 | rabbit |
| Smad3 | Cell Signaling Technology | 9523 | rabbit |
| Smad3 - phospho - Ser423/Ser425 | Cell Signaling Technology | 9520 | rabbit |
| Smad4 | Cell Signaling Technology | 9515 | rabbit |
| SOX17 | Sigma | SAB3300093 | rabbit |
| SOX9 | Sigma | HPA001758 | rabbit |

**Supplementary Table 2: Mutations of selected Wnt/Ras pathway component as determined by amplicon sequencing of frequently mutated loci in CRC**

| **Gene** | **Line 004** | **Line 007** | **Line 010** | **Line 015** | **Line 019** |
|---|---|---|---|---|---|
| KRAS | | G12D | G13D | G13D | A146T |
| NRAS | Q61L | | | | |
| PIK3CA | Q546P | | | | |
| AKT1 | | E17K | | | |
| PTEN | | | | T160I | |
| ARID1A | | | | | W1844* |
| APC | | R564*/ R60* | | | Q1378* |
| CTNNB1 | | | | G34V | |
| FBXW7 | | | | R465C | |
| TCF7L2 | | | | R465X | |

**Supplementary Table 3: Primer for quantitative PCR**

| Target gene | Species | Forward Primer Sequence | Reverse Primer Sequence |
|---|---|---|---|
| GAPDH | human | agccacatcgctcagacac | gcccaatacgaccaaatcc |
| AXIN1 | human | atggagctctccgagacaga | tagtacgccacaacgatgct |
| AXIN2 | human | agagcagctcagcaaaaagg | ccttcatacatcgggagcac |
| LGR5 | human | ttcccagggagtggattctat | accagactatgcctttggaaac |
| ASCL2 | human | gcaccaacacttggagatttt | aatggattctctgtgcccttag |
| CD44 | human | caagcaggaagaaggatggat | aacctgtgtttggatttgcag |
| EPHB2 | human | agcggcaagatgtacttcca | ggagccgatgatgagtgg |
| MAP2K1 | human | ttttaggaaaagttagcattgctgt | agggcttgacatctctgtgc |
| MAP2K2 | human | ttgaactcctggactatattgtgaac | aagtcggggggtgaacaca |
| MAPK3 | human | ccctagcccagacagacatc | Gcacagtgtccattttctaacagt |
| MAPK1 | human | caaagaactaatttttgaagagactgc | tcctctgagcccttgtcct |
| APC | human | gcatggaccaggacaaaaat | gaacacacacagcaggacagat |
| CTNNB1 | human | gctttcagttgagctgacca | caagtccaagatcagcagtctc |
| EGR1 | human | agccctacgagcacctgac | ggtttggctggggtaactg |
| SDHA | mouse | tgttcagttccaccccaca | tctccacgacacccttctgt |
| AXIN2 | mouse | aggatgctgaaggctcaaag | tcgccttcttgaaataatacctg |
| LGR5 | mouse | cttcactcggtgcagtgct | gatcagccagctaccaaatagg |
| ASCL2 | mouse | gagagctaagcccgatgga | aggtccaccaggagtcacc |
| BIRC5 | mouse | cccgatgacaacccgata | catctgcttcttgacagtgagg |
| EPHB2 | mouse | tagacatgccttgcacaacc | gtctcgttgacgctggagat |
| CCDN1 | mouse | tttctttccagagtcatcaagtgt | tgactccagaagggcttcaa |

**Supplementary Table 4: List of antibodies**

| Antibody | Company | Catalogue # | Species | Dilution |
|---|---|---|---|---|
| AXIN2 (76G6) | Cell Signaling Technology | #2151 | rabbit | 1 : 1000 |
| Vinculin | Millipore | AB6039 | rabbit | 1 : 1000 |
| Phospho-p90RSK (Ser380) | Cell Signaling Technology | #9341 | rabbit | 1 : 1000 |
| RSK1/RSK2/RSK3 (D7A2H) | Cell Signaling Technology | #14813 | rabbit | 1 : 1000 |
| p44/42 MAPK (Erk1/2) | Cell Signaling Technology | #9102 | rabbit | 1 : 1000 |
| Phospho-p44/42 MAPK (Erk1/2) (Thr202/Tyr204) | Cell Signaling Technology | #4370 | rabbit | 1 : 1000 |
| AXIN1 (C76H11) | Cell Signaling Technology | #2087 | rabbit | 1 : 1000 |
| beta-actin | Santa Cruz Biotechnology | 47778 | rabbit | 1 : 40.000 |
| beta-actin HRP | Santa Cruz Biotechnology | 47778 | rabbit | 1 : 250.000 |
| beta-catenin | BD Biosciences | 610154 | mouse | 1 : 2000 |
| GSK-3beta | BD Biosciences | 610201 | mouse | 1 : 1000 |
| APC (ALI 12-28) | Santa Cruz | 53165 | mouse | 1 : 1000 |
| EGR1 | Cell Signaling Technology | 15F7 | rabbit | 1 : 1000 |
| IgG | Cell Signaling Technology | 2729 | rabbit | |
| IgG1 K | eBioscience | 16-4714-81 | mouse | |

**Supplementary Table 5: Sequences of siRNA reagents (related to Methods).** siRNA reagents used for combinatorial knockdowns are highlighted by bold letters.

| Target | Company and catalogue # | siRNA # | Sequence |
|---|---|---|---|
| MAPK1 | MU-003555-04-0002 SMARTpool single sequences, GE health science | #1 | CCAAAGCUCUGGACUUAUU |
| | | **#2** | **AAACAGAUCUUUACAAGCU** |
| | | #3 | CAAGAGGAUUGAAGUAGAA |
| | | #4 | GUACAGGGCUCCAGAAAUU |
| MAPK3 | MU-003592-01-0002 SMARTpool single sequences GE health science | #1 | GACCGGAUGUUAACCUUUA |
| | | **#2** | **AGACUGACCUGUACAAGUU** |
| | | #3 | GCUACACGCAGUUGCAGUA |
| | | #4 | CCGGCUUCCUGACGGAGUA |
| MAP2K1 | MU-003571-01-0002 SMARTpool single sequences GE health science | **#1** | **GCACAUGGAUGGAGGUUCU** |
| | | #2 | GCAGAGAGAGCAGAUUUGA |
| | | #3 | GAGCAGAUUUGAAGCAACU |
| | | #4 | CCAGAAAGCUAAUUCAUCU |
| MAP2K2 | MU-003573-03-0002 SMARTpool single sequences GE health science | #1 | CAAAGACGAUGACUUCGAA |
| | | #2 | GAUCAGCAUUUGCAUGGAA |
| | | **#3** | **GGAAGCUGAUCCACCUUGA** |
| | | #4 | GAAAGUCAGCAUCGCGGUU |
| AXIN1 | MU-009625-01-0002 SMARTpool single sequences GE health science | #2 | GAAAGUGAGCGACGAGUUU |
| | | #3 | GAGCAAGUUUCACCGAAGA |
| | | #5 | CGAGAGCCAUCUACCGAAA |
| CTNNB1 | s438 (Ambion) | | CUGUUGGAUUGAUUCGAAAtt |
| APC | s1433 (Ambion) | | GGAUCUGUAUCAAGCCGUUtt |
| Scrambled | s29712 Silencer Select Negative Control (Ambion) | | |
