# Supplementary material for: MEK inhibitors activate Wnt signalling and induce stem cell plasticity in colorectal cancer
Source: Nat Commun. 2019 May 16;10:2197. doi: 10.1038/s41467-019-09898-0 (PMC6522484; doi:10.1038/s41467-019-09898-0)
Supplement: Supplementary file 3 — Description of Additional Supplementary Files [file 41467_2019_9898_MOESM3_ESM.docx]

**Description of Supplementary Files**

**File Name:** Supplementary Data 1

**Description:** Library composition and results of the exploratory compound screen. CellTiter-Glo and TCF-Wnt reporter signals for each compound were normalized to the median signal of all samples of the respective assay plate. Values are presented separately for each biological replicate and cell line.

**File Name:** Supplementary Data 2

**Description:** Library composition and results of the kinase inhibitor screen. CellTiter-Glo and TCF-Wnt reporter signals for each compound were normalized to the median signal of all samples of the respective assay plate. Values are presented separately for each biological replicate and cell line.
