# Supplementary material for: MEK inhibitors activate Wnt signalling and induce stem cell plasticity in colorectal cancer
Source: Nat Commun. 2019 May 16;10:2197. doi: 10.1038/s41467-019-09898-0 (PMC6522484; doi:10.1038/s41467-019-09898-0)
Supplement: Supplementary file 6 — Reporting Summary [file 41467_2019_9898_MOESM6_ESM.pdf]

nature research

Corresponding author(s): Michael Boutros

Last updated by author(s): Apr 3, 2019

Reporting Summary

Nature Research wishes to improve the reproducibility of the work that we publish. This form provides structure for consistency and transparency in reporting. For further information on Nature Research policies, see Authors & Referees and the Editorial Policy Checklist.

Statistics

For all statistical analyses, confirm that the following items are present in the figure legend, table legend, main text, or Methods section.

n/a	Confirmed	
☐	☒	The exact sample size (n) for each experimental group/condition, given as a discrete number and unit of measurement
☐	☒	A statement on whether measurements were taken from distinct samples or whether the same sample was measured repeatedly
☐	☒	The statistical test(s) used AND whether they are one- or two-sided<br>*Only common tests should be described solely by name; describe more complex techniques in the Methods section.*
☐	☒	A description of all covariates tested
☐	☒	A description of any assumptions or corrections, such as tests of normality and adjustment for multiple comparisons
☐	☒	A full description of the statistical parameters including central tendency (e.g. means) or other basic estimates (e.g. regression coefficient) AND variation (e.g. standard deviation) or associated estimates of uncertainty (e.g. confidence intervals)
☐	☒	For null hypothesis testing, the test statistic (e.g. F, t, r) with confidence intervals, effect sizes, degrees of freedom and P value noted<br>*Give P values as exact values whenever suitable.*
☒	☐	For Bayesian analysis, information on the choice of priors and Markov chain Monte Carlo settings
☒	☐	For hierarchical and complex designs, identification of the appropriate level for tests and full reporting of outcomes
☒	☐	Estimates of effect sizes (e.g. Cohen's d, Pearson's r), indicating how they were calculated

Our web collection on statistics for biologists contains articles on many of the points above.

Software and code

Policy information about availability of computer code

Data collection	N/A
Data analysis	Computer codes to reproduce all analysis involving gene expression and Digital Western Blot in this study is available at https://github.com/boutroslab/Supplemental-Material

For manuscripts utilizing custom algorithms or software that are central to the research but not yet described in published literature, software must be made available to editors/reviewers. We strongly encourage code deposition in a community repository (e.g. GitHub). See the Nature Research guidelines for submitting code & software for further information.

Data

Policy information about availability of data

All manuscripts must include a data availability statement. This statement should provide the following information, where applicable:

- Accession codes, unique identifiers, or web links for publicly available datasets
- A list of figures that have associated raw data
- A description of any restrictions on data availability

Expression data of microarrays is accessible through GEO under the accession number GSE114061 (associated Figures 7C, 8A, 8B, 8D)

Field-specific reporting

Please select the one below that is the best fit for your research. If you are not sure, read the appropriate sections before making your selection.

☒ Life sciences ☐ Behavioural & social sciences ☐ Ecological, evolutionary & environmental sciences

Life sciences study design

All studies must disclose on these points even when the disclosure is negative.

Sample size	No sample size estimation was performed for experiments involving cancer cell lines, organoids and mouse experiments. For xenograft experiments using colorectal cancer organoids, sample size was calculated based on previous publications in the literature on the effect size of the used drugs.
Data exclusions	No data exclusions
Replication	For all experiments, at least two independent biological replicates were performed. The number of replicates is presented in each figure legend.
Randomization	No randomization was performed for experiments involving cancer cell lines, organoids and mouse experiments. For xenograft experiments using colorectal cancer organoids, engrafted mice were randomly allocated to either treatment or placebo group.
Blinding	No blinding was performed for experiments involving cancer cell lines, organoids and mouse experiments. For xenograft experiments using colorectal cancer organoids, measurement of tumor size in engrafted mice was performed by a technician blinded regarding the drug treatment.

Reporting for specific materials, systems and methods

We require information from authors about some types of materials, experimental systems and methods used in many studies. Here, indicate whether each material, system or method listed is relevant to your study. If you are not sure if a list item applies to your research, read the appropriate section before selecting a response.

Materials & experimental systems

n/a	Involved in the study	
☐	☒	Antibodies
☐	☒	Eukaryotic cell lines
☒	☐	Palaeontology
☐	☒	Animals and other organisms
☒	☐	Human research participants
☒	☐	Clinical data

Methods

n/a	Involved in the study	
☒	☐	ChIP-seq
☒	☐	Flow cytometry
☒	☐	MRI-based neuroimaging

Antibodies

Antibodies used	A list of antibodies used in this study can be found in Supplementary Table 4
Validation	All antibodies were commercially purchased and references stating the validity of the antibody can be found on the respective web pages of the providers. Furthermore, correct size of bands were checked with previously published literature.

Eukaryotic cell lines

Policy information about cell lines

Cell line source(s)	HCT116, HT29, SW480, DLD1, HEK293T cells were derived from ATCC and SW403 from DSMZ
Authentication	Cell lines were obtained from ATCC and authentication of genotypes was performed by SNP profiling (Multiplexion, Heidelberg)
Mycoplasma contamination	Absence of mycoplasma infection was confirmed by regular testing.
Commonly misidentified lines (See ICLAC register)	N/A

Animals and other organisms

Policy information about studies involving animals; ARRIVE guidelines recommended for reporting animal research

Laboratory animals	C57BL/6 mice (6 months of age, 5 male and 5 female animals)<br>NOD SCID gamma (NSG) mice (6-7 weeks old, female, recruited from the Center for Preclinical Research, DKFZ, 28 mice)

Wild animals	N/A
Field-collected samples	N/A
Ethics oversight	Local Governmental Committee for Animal Experimentation<br>(RP Karlsruhe, Germany, license G-148/18 to T.Z. and K.M-D. / German Cancer Research Center)<br>(RP Karlsruhe, Germany, license G-176/12, G-146/15, G-188/18 to E.B. / Medical Faculty Mannheim, University of Heidelberg)

Note that full information on the approval of the study protocol must also be provided in the manuscript.
