# Supplementary material for: MEK inhibitors activate Wnt signalling and induce stem cell plasticity in colorectal cancer
Source: Nat Commun. 2019 May 16;10:2197. doi: 10.1038/s41467-019-09898-0 (PMC6522484; doi:10.1038/s41467-019-09898-0)
Supplement: Supplementary file 7 — Source Data [file 41467_2019_9898_MOESM7_ESM.zip › 172892_2_related_ms_3666553_pp6vx3.pdf]

Source Data

MEK inhibitors activate Wnt signalling and induce stem cell plasticity in colorectal cancer

Zhan et al.

Fig3E

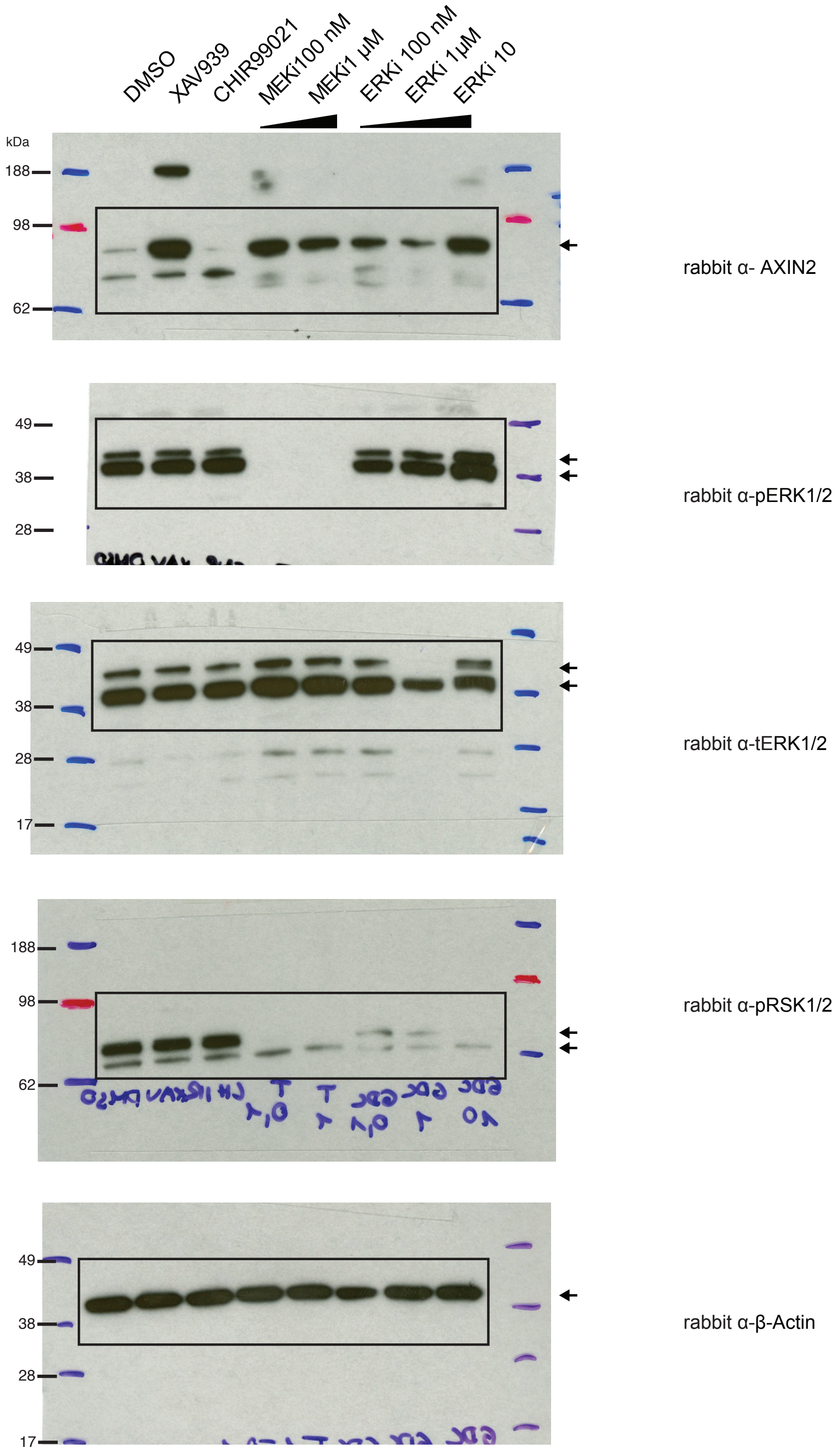
DMSO
XAV939
CHIR99021
MEKi100 nM
MEKi1 µM
ERKi 100 nM
ERKi 1µM
ERKi 10
kDa
188
98
62
rabbit α- AXIN2
49
38
28
rabbit α-pERK1/2
49
38
28
17
rabbit α-tERK1/2
188
98
62
rabbit α-pRSK1/2
49
38
28
17
rabbit α-β-Actin

Fig4C

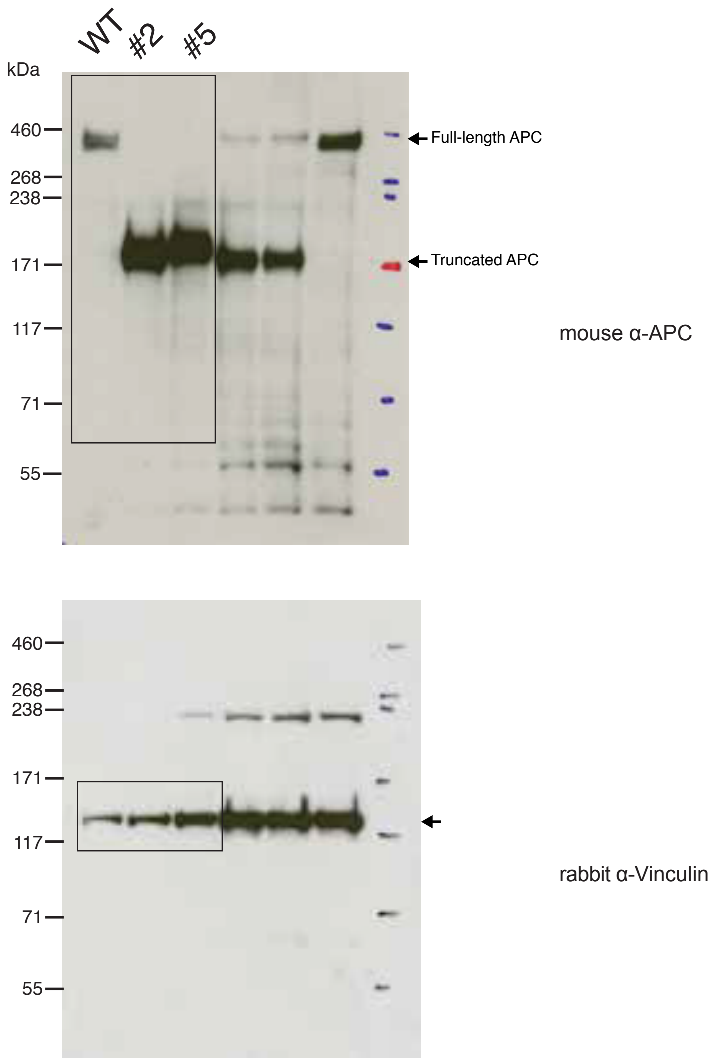
WT
#2
#5
kDa
460
268
238
171
117
71
55
Full-length APC
Truncated APC
mouse α-APC
460
268
238
171
117
71
55
rabbit α-Vinculin

Fig.5B

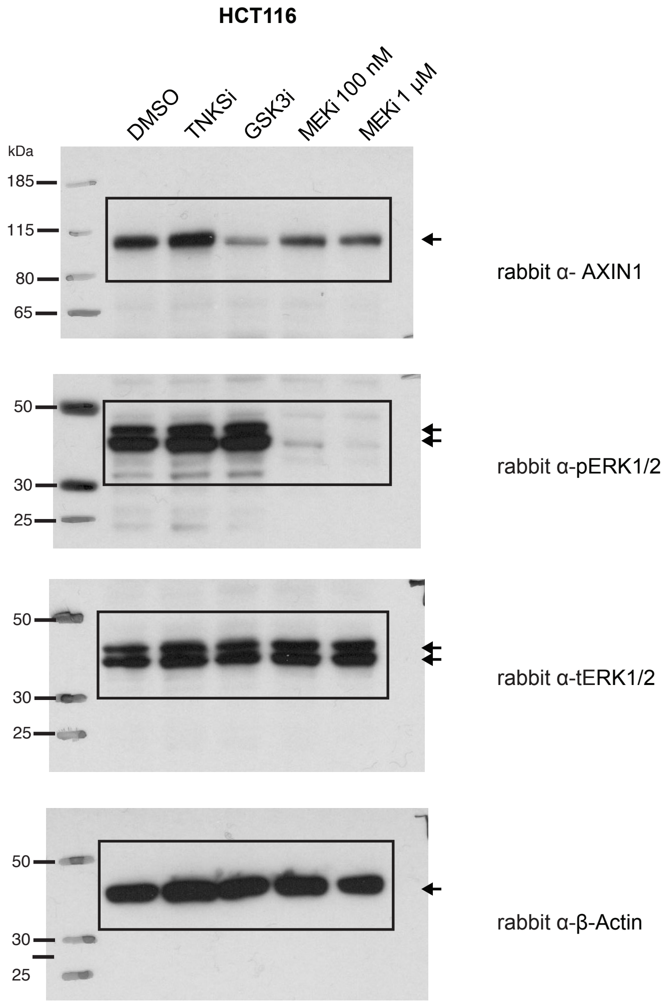
HCT116
DMSO
TNKSi
GSK3i
MEKi 100 nM
MEKi 1 µM
kDa
185
115
80
65
rabbit α- AXIN1
50
30
25
rabbit α-pERK1/2
50
30
25
rabbit α-tERK1/2
50
30
25
rabbit α-β-Actin

Fig5C

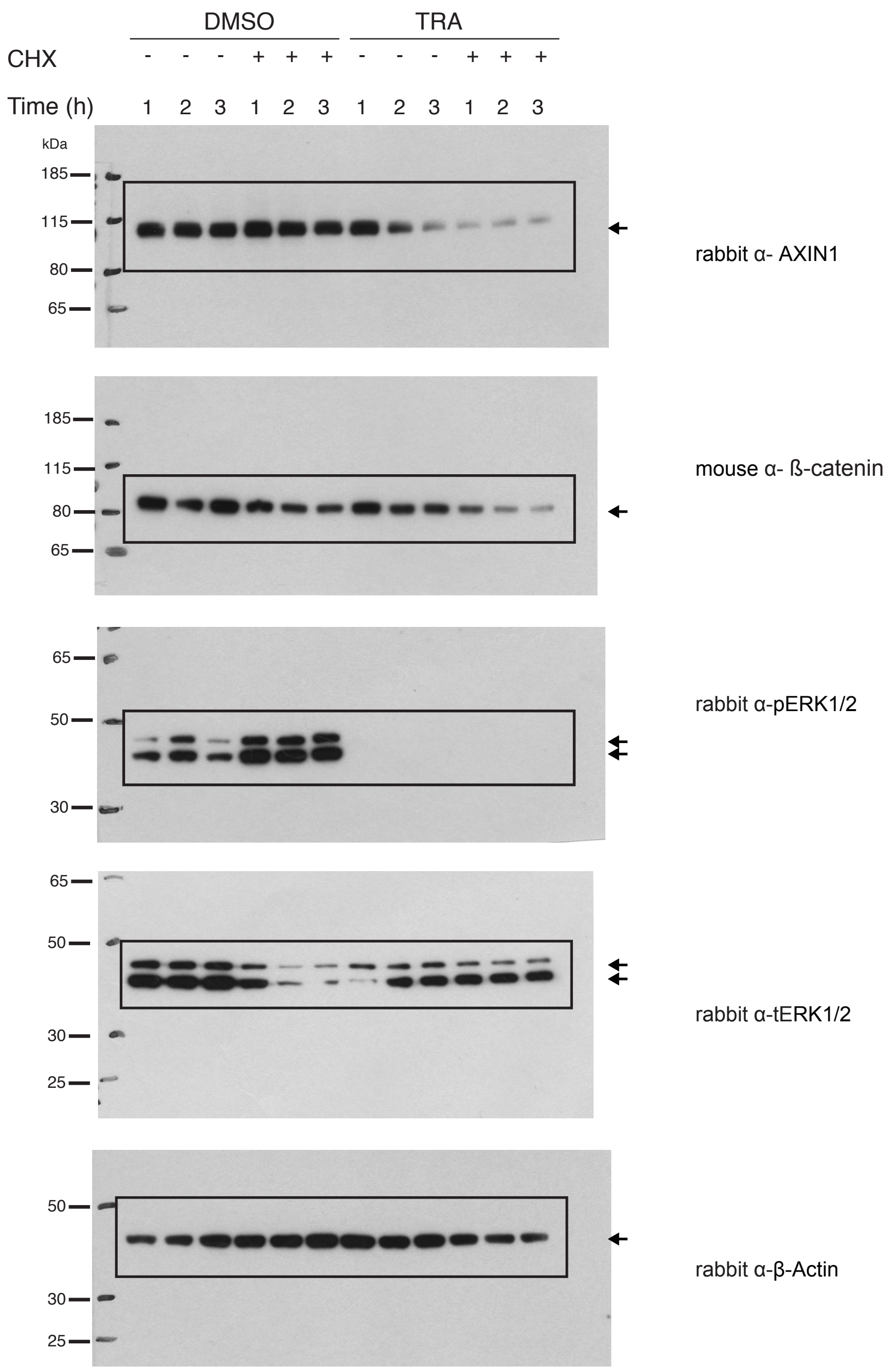
DMSO
TRA
CHX
- - - + + + - - - + + +
Time (h)
1 2 3 1 2 3 1 2 3 1 2 3
kDa
185
115
80
65
rabbit α- AXIN1
185
115
80
65
mouse α- ß-catenin
65
50
30
rabbit α-pERK1/2
65
50
30
25
rabbit α-tERK1/2
50
30
25
rabbit α-β-Actin

Fig5F

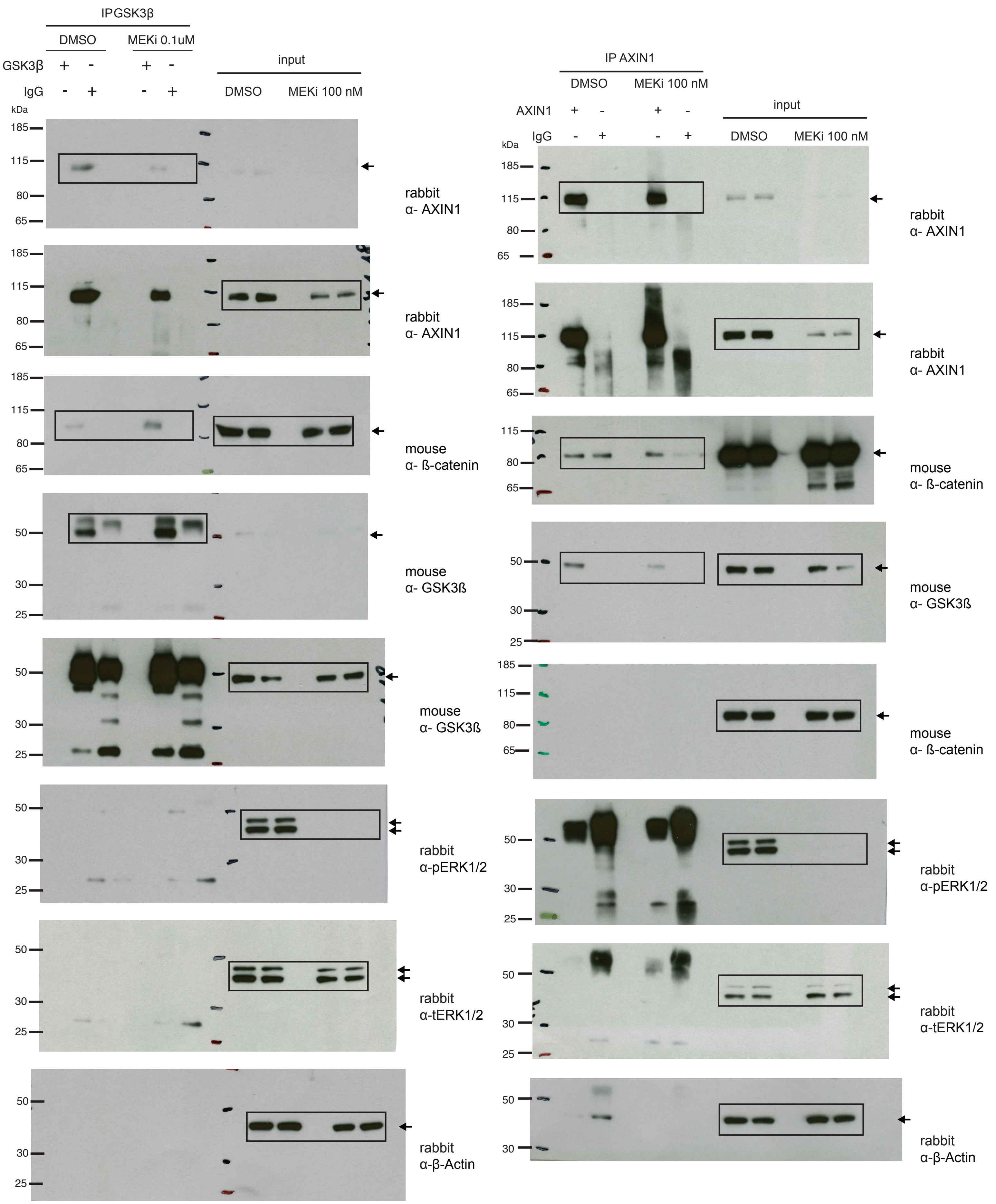

IPGSK3β
DMSO
MEKi 0.1uM
GSK3β + - + -
IgG - + - +
input
DMSO
MEKi 100 nM
kDa
185
115
80
65
50
30
25
rabbit α- AXIN1
rabbit α- AXIN1
mouse α- ß-catenin
mouse α- GSK3ß
mouse α- GSK3ß
rabbit α-pERK1/2
rabbit α-tERK1/2
rabbit α-β-Actin
IP AXIN1
DMSO
MEKi 100 nM
AXIN1 + - + -
IgG - + - +
input
DMSO
MEKi 100 nM
kDa
rabbit α- AXIN1
rabbit α- AXIN1
mouse α- ß-catenin
mouse α- GSK3ß
mouse α- ß-catenin
rabbit α-pERK1/2
rabbit α-tERK1/2
rabbit α-β-Actin

Fig7D

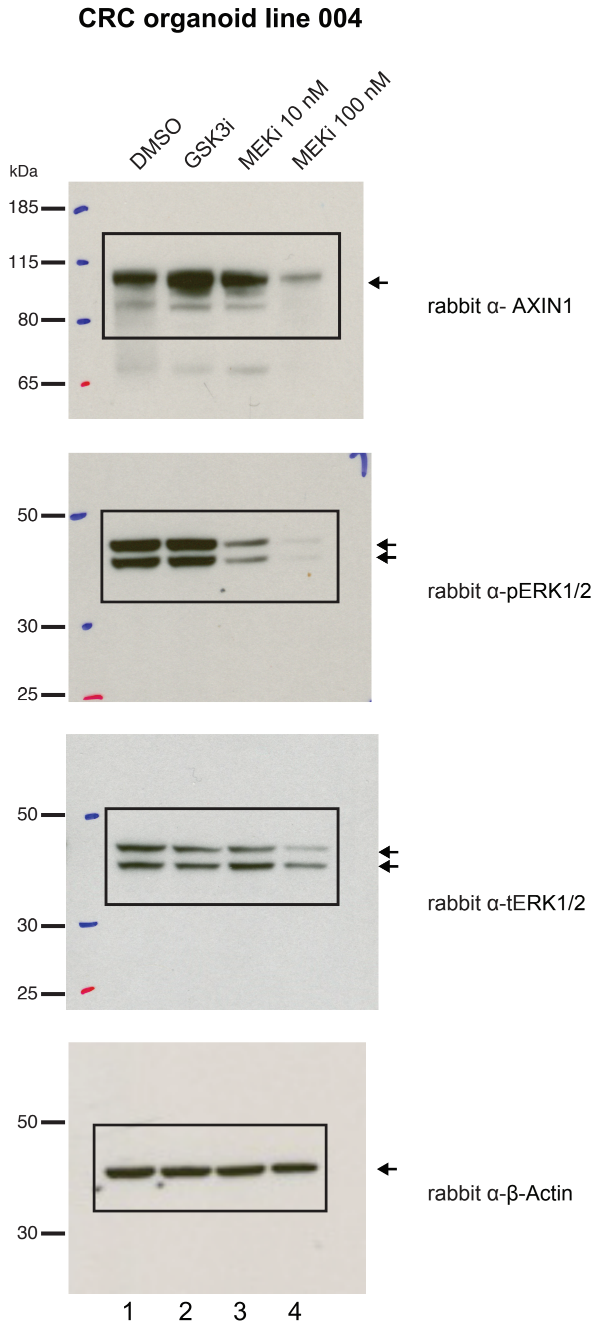
CRC organoid line 004
DMSO
GSK3i
MEKi 10 nM
MEKi 100 nM
kDa
185
115
80
65
rabbit α- AXIN1
50
30
25
rabbit α-pERK1/2
50
30
25
rabbit α-tERK1/2
50
30
rabbit α-β-Actin
1
2
3
4

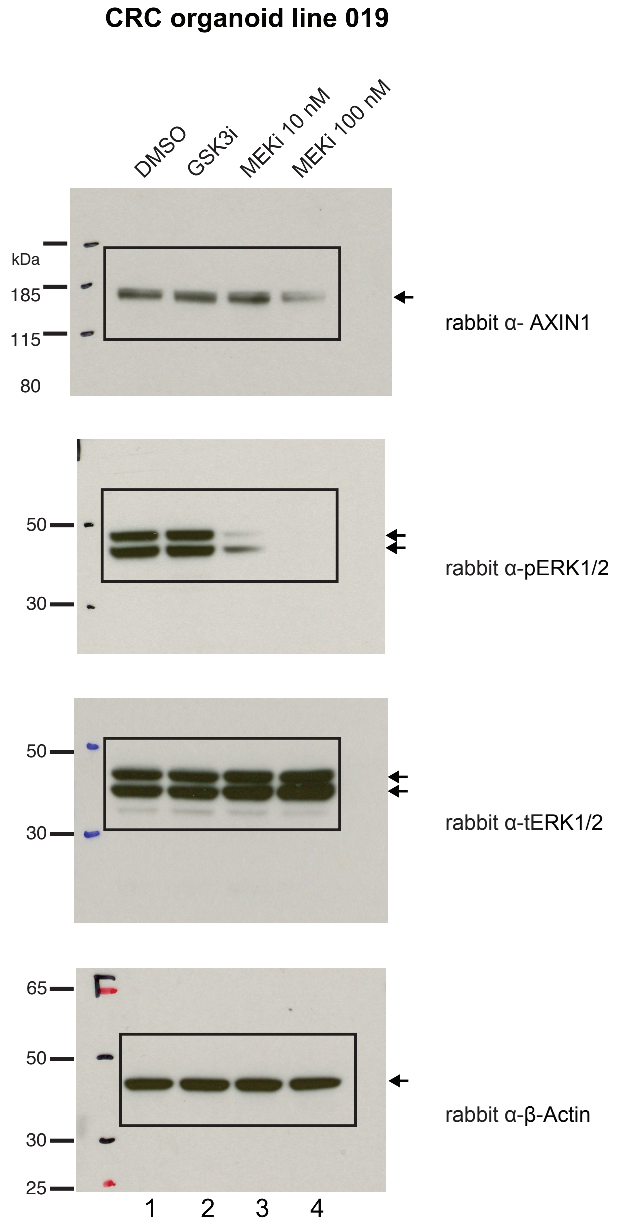
CRC organoid line 019
DMSO
GSK3i
MEKi 10 nM
MEKi 100 nM
kDa
185
115
80
rabbit α- AXIN1
50
30
rabbit α-pERK1/2
50
30
rabbit α-tERK1/2
65
50
30
25
rabbit α-β-Actin
1
2
3
4

FigS2B

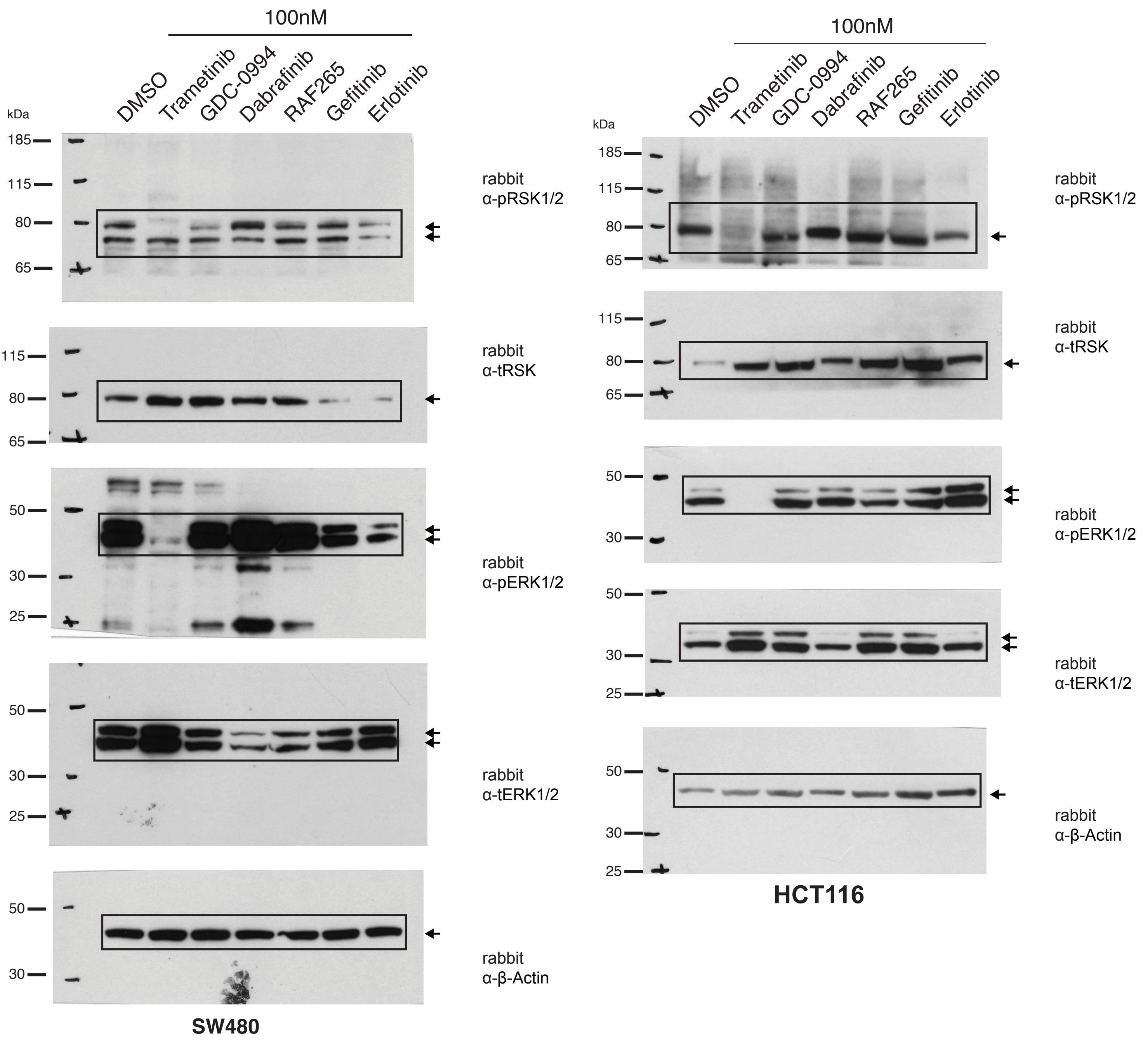
100nM
DMSO
Trametinib
GDC-0994
Dabrafinib
RAF265
Gefitinib
Erlotinib
kDa
185
115
80
65
50
30
25
rabbit α-pRSK1/2
rabbit α-tRSK
rabbit α-pERK1/2
rabbit α-tERK1/2
rabbit α-β-Actin
SW480
HCT116

FigS6A

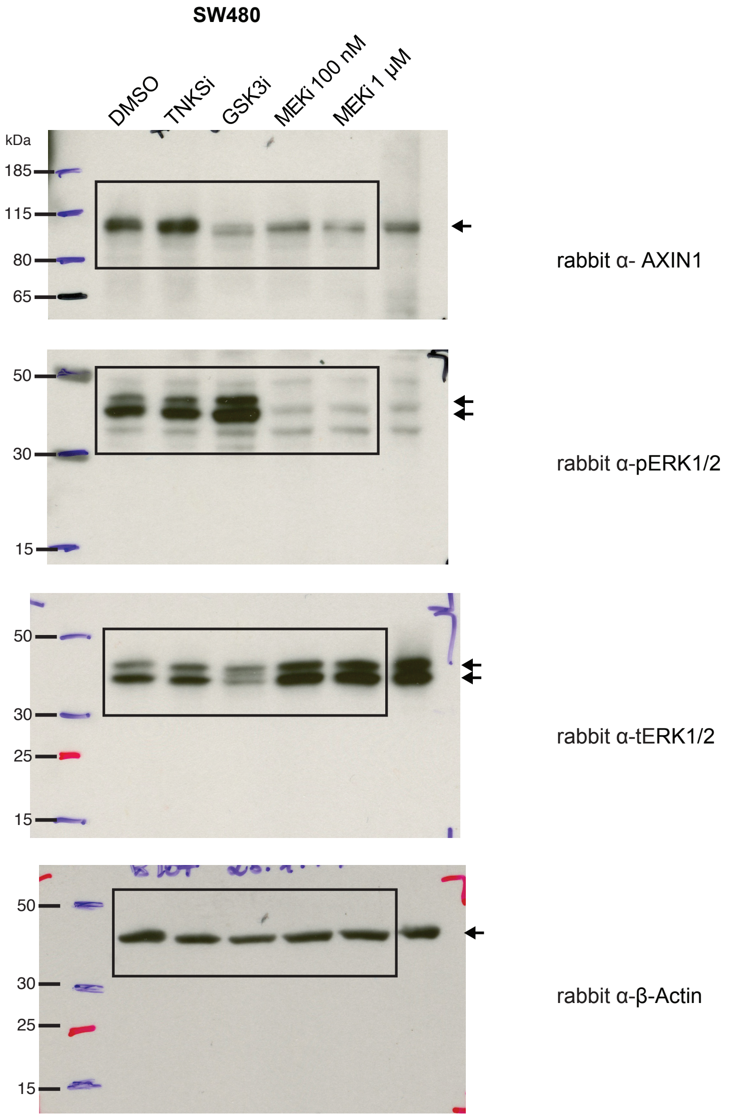
SW480
DMSO
TNKSi
GSK3i
MEKi 100 nM
MEKi 1 µM
kDa
185
115
80
65
rabbit α- AXIN1
50
30
15
rabbit α-pERK1/2
50
30
25
15
rabbit α-tERK1/2
50
30
25
15
rabbit α-β-Actin

FigS6D

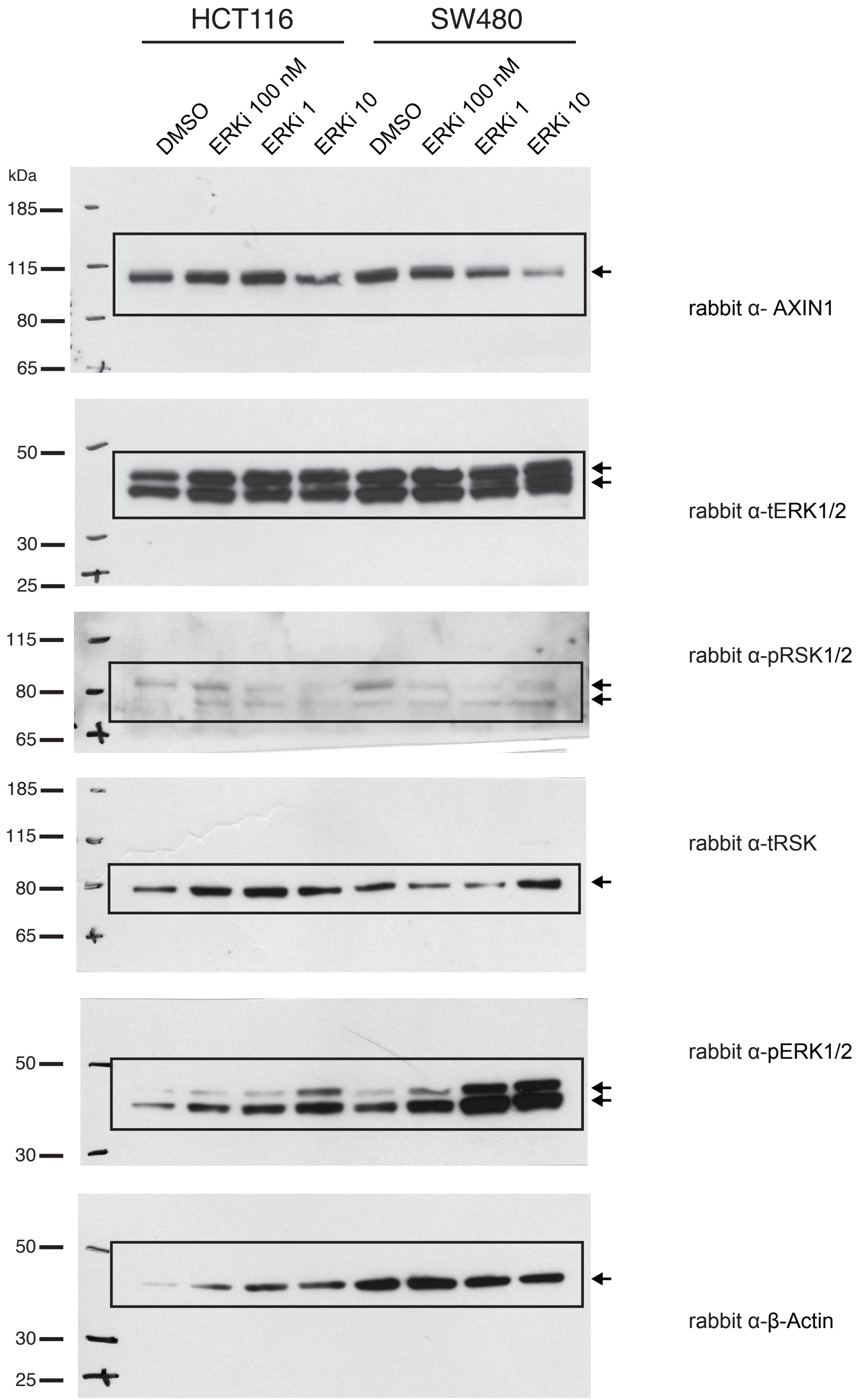
HCT116
SW480
DMSO
ERKi 100 nM
ERKi 1
ERKi 10
DMSO
ERKi 100 nM
ERKi 1
ERKi 10
kDa
185
115
80
65
rabbit α- AXIN1
50
30
25
rabbit α-tERK1/2
115
80
65
rabbit α-pRSK1/2
185
115
80
65
rabbit α-tRSK
50
30
rabbit α-pERK1/2
50
30
25
rabbit α-β-Actin

FigS6E

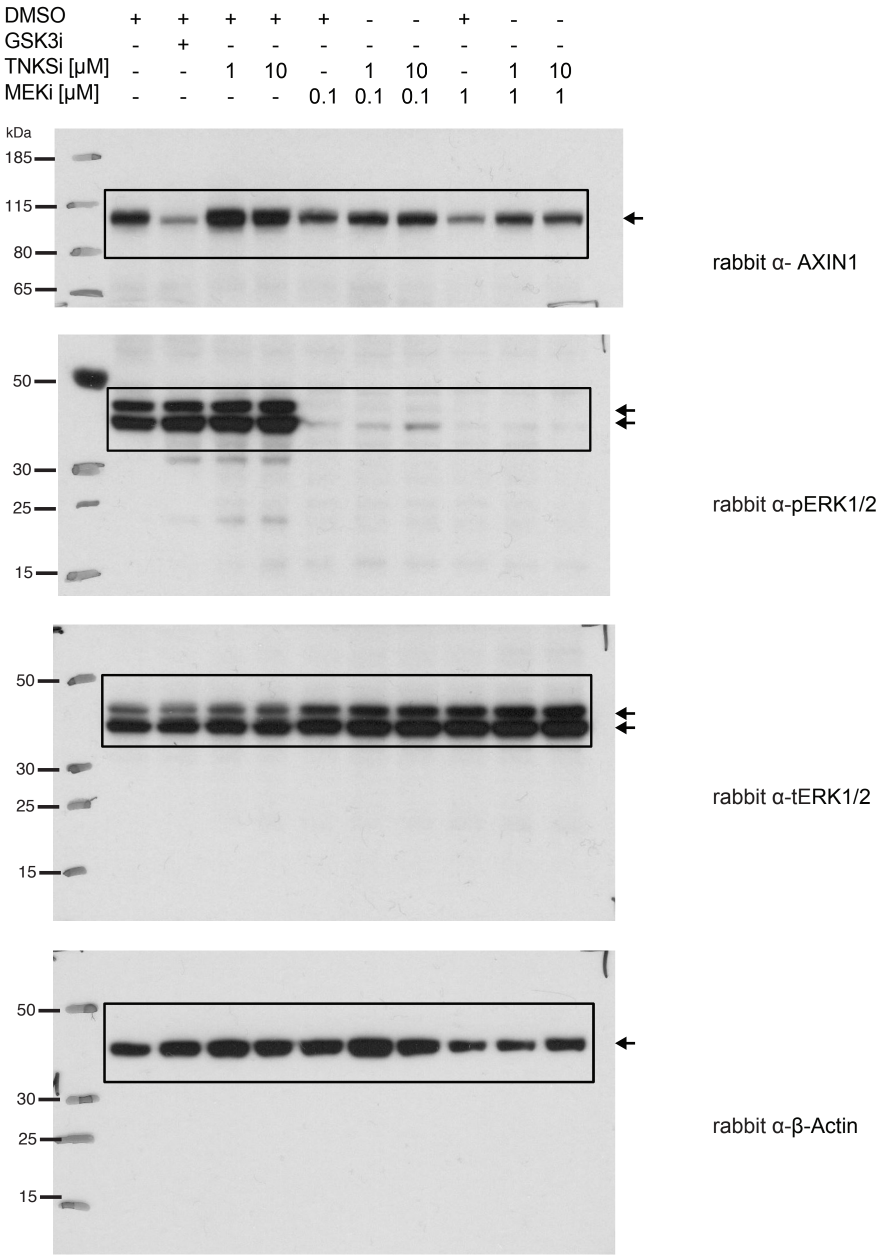
DMSO + + + + + - - + - -
GSK3i - + - - - - - - - -
TNKSi [µM] - - 1 10 - 1 10 - 1 10
MEKi [µM] - - - - 0.1 0.1 0.1 1 1 1
kDa
185
115
80
65
rabbit α- AXIN1
50
30
25
15
rabbit α-pERK1/2
50
30
25
15
rabbit α-tERK1/2
50
30
25
15
rabbit α-β-Actin

FigS6F

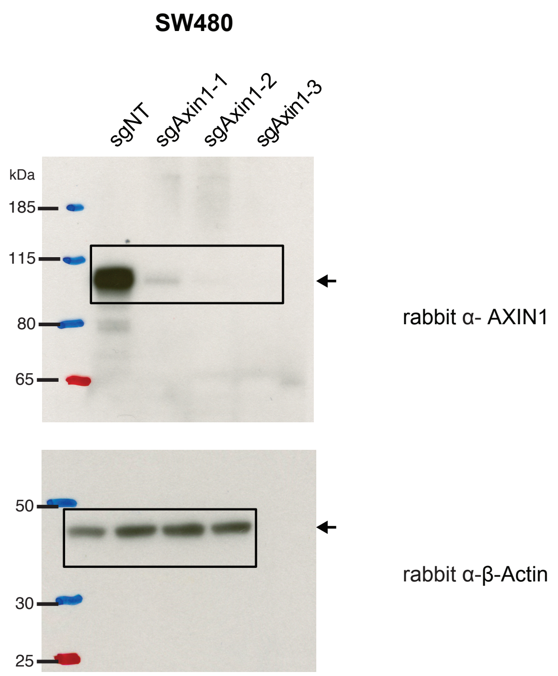
SW480
sgNT
sgAxin1-1
sgAxin1-2
sgAxin1-3
kDa
185
115
80
65
rabbit α- AXIN1
50
30
25
rabbit α-β-Actin

FigS7B

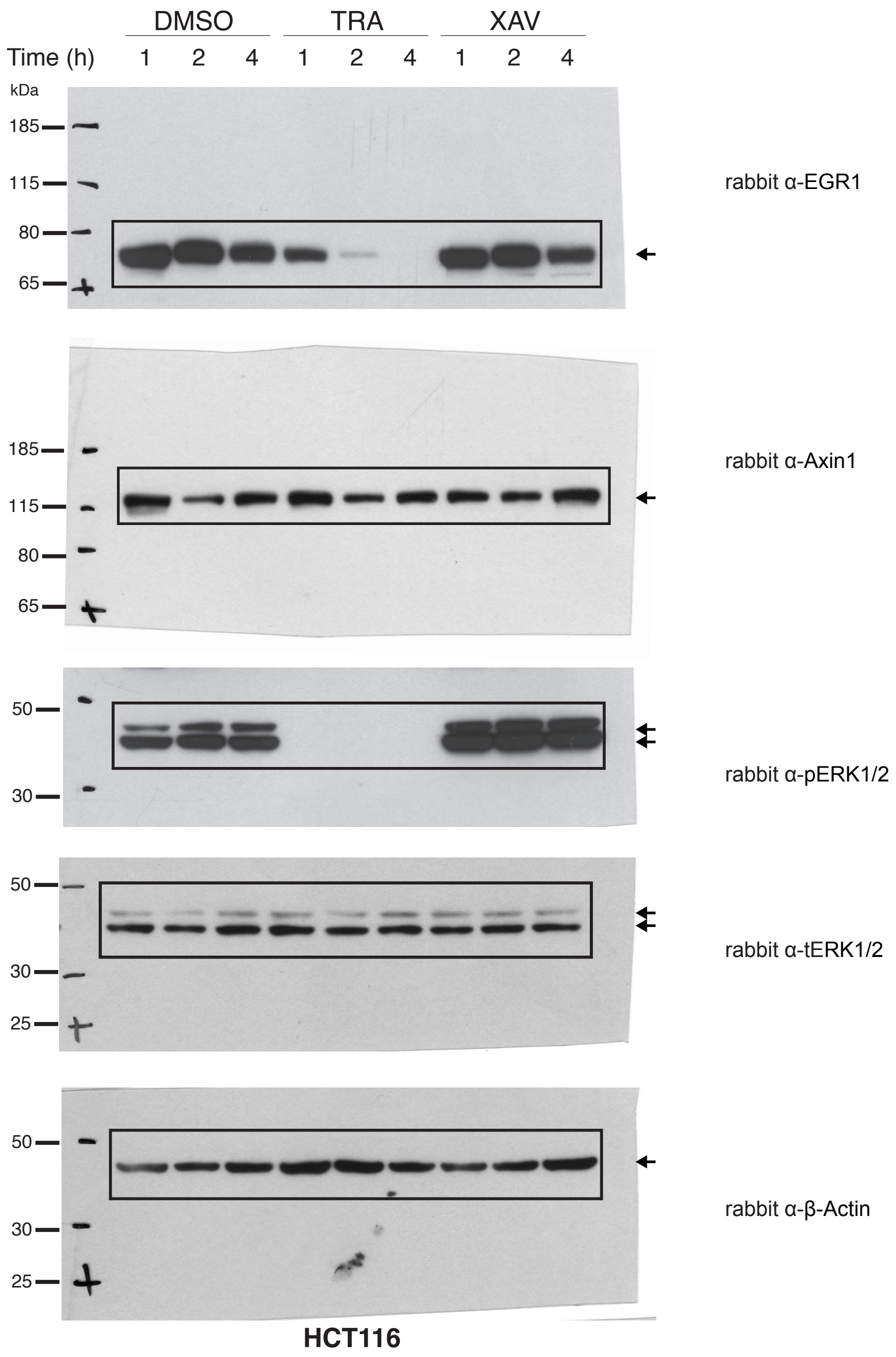
DMSO
TRA
XAV
Time (h)
1 2 4 1 2 4 1 2 4
kDa
185
115
80
65
rabbit α-EGR1
185
115
80
65
rabbit α-Axin1
50
30
rabbit α-pERK1/2
50
30
25
rabbit α-tERK1/2
50
30
25
rabbit α-β-Actin
HCT116
